# Inter‐ and Intra‐Rater Reliability of Myotonometric Assessment of the Mechanical Properties of Caesarean Section Scar Skin Using the MyotonPRO With an L‐Shaped Probe

**DOI:** 10.1111/srt.70315

**Published:** 2026-01-09

**Authors:** Monika Nosiadek, Jarosław Marusiak, Karolina Gamoń, Dawid Bączkowicz

**Affiliations:** ^1^ Fizjoterapia Monika Nosiadek Rybnik Poland; ^2^ Division of Kinesiology Department of Physiotherapy in Movement System Dysfunctions and Kinesiology Faculty of Physiotherapy Wroclaw University of Health and Sport Science Wroclaw Poland; ^3^ Faculty of Physical Education and Physiotherapy Opole University of Technology Opole Poland; ^4^ Voivodship Specialist Hospital No. 3 in Rybnik Rybnik Poland

**Keywords:** elasticity, measurement stability, myotonometry, tone, stiffness

## Abstract

**Background:**

Myotonometry is a noninvasive method for assessing the mechanical properties of soft tissues, including muscles and skin. The L‐shaped probe, designed specifically for skin assessment, may be useful for evaluating caesarean section (C‐section) scars. However, reliability data for MyotonPRO measurements of C‐section scars with this probe are limited. This study aimed to evaluate the inter‐rater and intra‐rater reliability of measuring the mechanical properties of C‐section scar skin using the MyotonPRO with the L‐shaped probe.

**Materials and Methods:**

Twenty‐five women (23–42 years) with C‐section scars were examined. Two raters conducted the myotonometric assessments over two consecutive days. Measurements were taken with the participant supine, using the L‐shaped probe at six points around the scar (three above, three below; positioned at the scar's endpoints and centre), each tested in three directions (right, left and upward/downward). Reliability was analysed using the intraclass correlation coefficient (ICC) with 95% confidence intervals (CIs), the standard error of measurement (SEM), minimal detectable change (MDC), coefficient of variation (CV) and Bland–Altman plots.

**Results:**

Inter‐rater reliability was good to excellent (ICC = 0.75–0.90), while intra‐rater reliability was moderate to good, with slightly lower ICCs for consecutive‐day measurements. SEM, MDC and CV values supported these findings, showing lower measurement error and narrower variability ranges between raters than within repeated measurements by the same rater.

**Conclusion:**

The MyotonPRO with the L‐shaped probe provides reliable inter‐ and intra‐rater measurements of C‐section scar skin mechanical properties, making it a valuable tool for scar evaluation.

## 1 Introduction

Myotonometry is a relatively novel, easy to perform and non‐invasive method to characterise the mechanical properties of superficial soft tissues [1]. It involves the application of an external, brief (15 ms), low‐intensity (0.58 N) mechanical impulse applied to the skin. The oscillatory tissue response allows for the immediate assessment of tension, stiffness and elasticity, among other parameters [2]. To date, myotonometry especially has been used to evaluate the mechanical properties of muscles [3, 4, 5], tendons [6, 7, 8] and ligaments [9], showing high specificity and moderate to excellent intra‐ and inter‐rater reliability [10, 11, 12, 13, 14, 15, 16, 17].

Myotonometry has also shown high reproducibility and repeatability in the assessment of mechanical properties of the skin [18, 19]. Öztürk et al. [20] indicated that myotonometric measurement of skin tone and stiffness is a reliable method that exhibits satisfactory functionality and correlation with the gold standard skin assessment methods. Furthermore, myotonometry has been used to assess post‐caesarean section scars, and the results were compared with those of unscarred skin [21, 22]. These studies have shown that scars modify the viscoelastic properties of the skin and subcutaneous tissues, with increased stiffness and/or decreased compliance and elasticity, based mainly on measurements using the standard cylindrical probe applied perpendicularly to the skin.

However, to assess skin mechanical properties, dedicated L‐shaped probes are recommended as more reliable than standard cylindrical probes for muscles, tendons and ligaments [18]. L‐shaped probes deliver impulses horizontally along the skin, inducing damped surface oscillations and enabling multidirectional pliability assessment, unlike cylindrical probes [18]. Nevertheless, the vast majority of studies assessing the mechanical properties of skin and scars have been conducted using a standard cylindrical probe [21, 22], despite the fact that the stiffness values recorded with this probe differ significantly from those obtained using L‐shaped probes [19]. Simultaneously, the skin surrounding the scar changes its mechanical properties, resulting in an overall reduction in the pliability of both the scar and the surrounding skin. The standard clinical evaluation of scar pliability typically relies on the Patient and Observer Scar Assessment Scale (POSAS) [23, 24], which assesses scar mobility by manual skin displacement and subsequent subjective scoring [25]. However, due to its subjective nature and dependence on rater experience, POSAS may lack inter‐ and intra‐rater consistency. Therefore, using the MyotonPRO with an L‐shaped probe could offer a more objective and reliable method for assessing scar skin pliability. However, to the best of our knowledge, data are lacking on the intra‐ and inter‐rater reliability of myotonometric assessments of the mechanical properties of caesarean section (C‐section) scar skin using the MyotonPRO with the L‐shaped probe. This knowledge is essential for further research evaluating the extent of scarring and determining treatment response [26].

Thus, this study aimed to evaluate the inter‐rater and intra‐rater reliability of measuring the mechanical properties of C‐section scar skin using the MyotonPRO with the L‐shaped probe. It was hypothesised that the reliability of the myotonometric assessment of scar skin mechanical properties would be good to excellent, with intraclass correlation coefficient (ICC) values above 0.75.

## 2 Methods

### 2.1 Participants

Twenty‐five healthy adult women aged 23–42 years after C‐section were included in the study (mean age 31 ± 4 years, height 165 ± 5 cm, weight 71 ± 14 kg, BMI 25.5 ± 5.8 kg/m^2^). The required sample size was determined a priori using a power analysis for an intraclass ICC hypothesis test, set to achieve 80% power to detect an expected ICC of 0.90, with a minimum acceptable ICC of 0.75, at a two‐tailed significance level of 0.05. The analysis indicated a need for 22 participants; allowing for an anticipated 10% dropout, the target was increased to 25 to ensure robust reliability estimates.

The study recruited women who were receiving treatment at the private physiotherapy practice “Fizjoterapia Monika Nosiadek” in Rybnik, Poland.

The inclusion criteria for participation in the study were as follows: (i) presence of a scar after the first C‐section, (ii) age of the scar between 6 and 7 weeks after the C‐section (after the postpartum period) and (iii) a completely healed scar. The study excluded women with a non‐standard C‐section shape and those with failure of the scar healing process (e.g., due to infection). Ethical approval was obtained from the Bioethics Committee of the Medical University of Wrocław, Poland (no. 2022–3351). All participants signed an informed consent form prior to enrolment.

### 2.2 Myotonometric Instrumentation

**FIGURE 1:**
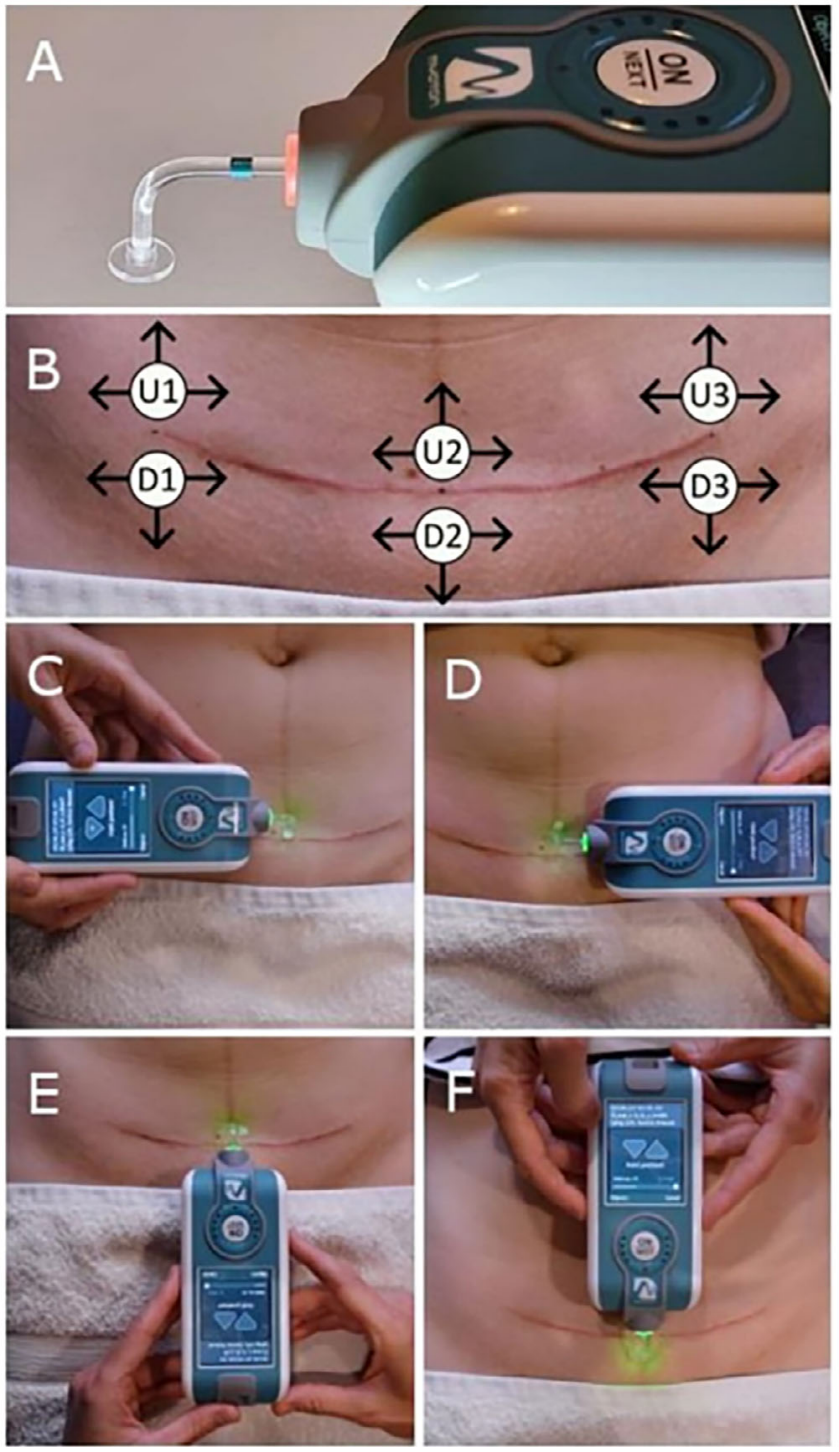
MyotonPRO device with an L‐shaped probe (A); three measurement points up the scar (U1–U3) and down the scar (D1–D3), with testing directions indicated by arrows (B); assessment of the mechanical properties of the caesarean scar in four directions: right (C), left (D), cranial (E) and sacral (F).

Myotonometric measurements of scar skin mechanical properties were performed using the MyotonPRO device (Myoton AS, Tallinn, Estonia), which is compact (resembling a mobile phone in appearance and size), portable and easy to operate (Figure 1). The measurement is noninvasive and quick, taking between 3 and 30 s per test, depending on the selected option. The device consists of a body with a display and a section (at the bottom of the body) where the measurement probe is extended (Figure 1A, D–F). The myotonometer contains an electromagnetic actuator used to generate mechanical impulses that is connected to the measurement probe, an accelerometer mounted on the inner part of the probe, a processor and other electronic components. These components activate the actuator, process the accelerometer signal and calculate myotonometric parameters. In our experiment, we used a special L‐shaped probe (Figure 1A), which has been reported to be more appropriate for skin mechanical property measurements [18]. During myotonometric measurements, the tip of the shorter arm of the L‐shaped probe is attached to the skin using double‐sided adhesive tape (Figure 1A, C–F). The operating principle of the MyotonPRO device is simple. The myotonometer generates a brief, 15‐ms low‐force (0.58 N) mechanical impulse that is transmitted through the L‐shaped probe to the skin, inducing mechanical oscillations along the horizontal plane without triggering reflexive muscle contractions beneath the skin. An accelerometer attached to the inner part of the measurement probe measures the changes in acceleration during these skin oscillations.

### 2.3 Measurement Procedure

Two raters (R1 and R2) received training on the use of the MyotonPRO and the standardisation of the measurement procedure prior to data collection. The raters conducted measurements during two testing sessions on consecutive days: session 1 (S1) on the first day and session 2 (S2) on the second day. Myotonometric measurements of scar skin mechanical properties were conducted with the participant lying supine on a treatment table, as described by Gilbert et al. [21], in a room maintained at a constant temperature of 24°C. Myotonometric measurements were taken at six points on the skin adjacent to the scar: three points located cranially, that is, up the scar (designated as U1, U2 and U3) and three points located caudally, that is, down the scar (designated as D1, D2 and D3) (Figure 1B–F). These points were positioned along the line of the scar at the two endpoints (U1 and D1 on the left scar endpoint, and U3 and D3 on the right scar endpoint) and in the central part of the scar (U2 and D2) (Figure 1B). Each measurement point was located 1 cm from the scar, perpendicular to its long axis (Figure 1B). At each of the six measurement points, myotonometric measurements were performed in three directions from the scar: to the right and to the left (for all six points), and either upward (for measurement points up the scar: U1, U2 and U3) or downward (for measurement points down the scar: D1, D2 and D3), as shown in Figure 1B. Therefore, in Tables 1, 2, 3, 4, the myotonometric measurements in these directions are labelled as follows: R and L for the rightward and leftward directions (for all six measurement points), respectively, and U and D for the upward (for measurement points U1, U2 and U3) and downward directions (for measurement points D1, D2 and D3), respectively. Thus, each participant underwent 18 measurements, which were obtained by multiplying the six measurement points by the three directions measured at each point (Figure 1B). The measurements were conducted by positioning the testing probe and the myotonometer body relative to the scar surface, ensuring that the L‐shaped probe extended from the myotonometer body to displace the skin in the desired horizontal direction during mechanical impulse generation. For the upward measurements, the long arm of the testing probe (and the myotonometer body) was oriented caudally, with the short arm was oriented cranially (Figure 1B, E). Conversely, for the downward measurements, the long arm of the probe was oriented cranially, while the short arm was oriented caudally (Figure 1B, F). For rightward measurements, the long arm of the probe was oriented to the left, whereas the short arm was oriented to the right (Figure 1B, C). Conversely, for leftward measurements, the long arm of the probe was oriented to the right, with the short arm oriented to the left (Figure 1B, D).

**TABLE 1:** Results of the inter‐rater reliability of the MyotonPRO parameter values in session 1.

Reliability statistical metrics			Measurement points on the scar																
		U1			U2			U3			D1			D2			D3		
		L	U	R	L	U	R	L	U	R	L	D	R	L	D	R	L	D	R
F‐MYO	ICC	0.86	0.94	0.81	0.74	0.61	0.87	0.83	0.96	0.86	0.85	0.96	0.92	0.85	0.73	0.87	0.92	0.90	0.77
	LCI	0.73	0.89	0.65	0.53	0.35	0.76	0.68	0.92	0.73	0.72	0.93	0.84	0.71	0.52	0.76	0.85	0.81	0.59
	UCI	0.93	0.97	0.90	0.86	0.79	0.94	0.91	0.98	0.93	0.92	0.98	0.96	0.92	0.86	0.94	0.96	0.95	0.88
	SEM	0.50	0.69	0.49	0.73	1.80	0.51	0.58	0.59	0.46	0.47	0.51	0.38	0.63	1.16	0.50	0.50	0.83	0.78
	MDC	1.38	1.92	1.36	2.03	4.99	1.41	1.60	1.62	1.27	1.31	1.40	1.06	1.75	3.20	1.39	1.37	2.30	2.16
	CV	3.16	4.33	3.08	4.27	12.22	2.96	3.45	3.71	2.81	2.96	3.16	2.40	3.80	8.01	2.99	2.93	5.28	4.69
S‐MYO	ICC	0.86	0.91	0.72	0.84	0.89	0.88	0.88	0.95	0.83	0.86	0.87	0.90	0.83	0.87	0.81	0.88	0.96	0.73
	LCI	0.74	0.83	0.51	0.70	0.80	0.78	0.78	0.89	0.69	0.73	0.90	0.80	0.68	0.76	0.65	0.77	0.93	0.52
	UCI	0.93	0.95	0.85	0.92	0.95	0.94	0.94	0.97	0.91	0.93	0.98	0.95	0.91	0.94	0.90	0.94	0.98	0.86
	SEM	8.71	0.78	12.93	11.33	9.52	11.16	13.23	12.32	12.75	8.31	10.68	10.75	13.55	11.66	12.86	14.93	9.93	18.03
	MDC	24.15	39.86	35.84	31.41	26.39	30.94	36.67	34.16	35.33	23.03	32.31	29.79	37.56	32.31	35.65	41.39	27.51	49.96
	CV	3.91	5.83	5.45	4.48	4.51	4.28	5.20	4.89	5.33	3.71	4.61	4.52	5.52	5.60	5.07	5.74	4.16	7.36
D‐MYO	ICC	0.84	0.89	0.81	0.84	0.89	0.91	0.90	0.93	0.92	0.75	0.98	0.89	0.85	0.94	0.89	0.91	0.91	0.90
	LCI	0.70	0.78	0.65	0.71	0.78	0.82	0.82	0.87	0.84	0.55	0.96	0.79	0.72	0.87	0.80	0.83	0.83	0.81
	UCI	0.92	0.94	0.90	0.92	0.94	0.95	0.95	0.97	0.96	0.87	0.99	0.94	0.92	0.97	0.95	0.95	0.96	0.95
	SEM	0.10	0.12	0.09	0.10	0.12	0.09	0.09	0.10	0.09	0.13	0.06	0.08	0.10	0.10	0.10	0.08	0.12	0.10
	MDC	0.27	0.32	0.26	0.27	0.33	0.25	0.25	0.27	0.25	0.35	0.16	0.23	0.28	0.29	0.29	0.22	0.33	0.27
	CV	5.23	5.92	5.08	4.84	6.09	4.47	4.76	4.85	4.73	7.68	3.03	5.38	5.72	6.41	5.70	5.07	6.52	5.47
R‐MYO	ICC	0.86	0.94	0.80	0.85	0.90	0.89	0.89	0.94	0.86	0.91	0.90	0.87	0.80	0.88	0.85	0.88	0.95	0.73
	LCI	0.73	0.89	0.63	0.71	0.81	0.79	0.88	0.79	0.74	0.83	0.80	0.75	0.63	0.78	0.72	0.78	0.91	0.52
	UCI	0.93	0.97	0.90	0.92	0.95	0.94	0.94	0.97	0.93	0.96	0.95	0.93	0.89	0.94	0.92	0.94	0.98	0.86
	SEM	0.67	1.02	0.72	0.63	0.89	0.61	0.71	1.12	0.69	0.51	1.00	0.70	0.86	0.86	0.64	0.77	0.71	1.08
	MDC	1.87	2.83	1.98	1.74	2.47	1.70	1.98	3.09	1.92	1.41	2.76	1.93	2.39	2.38	1.78	2.13	1.96	2.99
	CV	3.19	4.88	3.52	3.27	3.83	3.24	3.74	5.33	3.43	2.45	4.66	3.50	4.44	3.72	3.38	4.18	3.37	5.52
C‐MYO	ICC	0.81	0.93	0.80	0.81	0.88	0.86	0.87	0.94	0.81	0.88	0.83	0.71	0.75	0.90	0.79	0.83	0.95	0.58
	LCI	0.65	0.87	0.63	0.66	0.78	0.74	0.75	0.88	0.66	0.78	0.69	0.49	0.55	0.82	0.62	0.68	0.90	0.30
	UCI	0.90	0.97	0.89	0.90	0.94	0.93	0.93	0.97	0.90	0.94	0.91	0.85	0.87	0.95	0.89	0.91	0.98	0.77
	SEM	0.04	0.06	0.04	0.04	0.06	0.04	0.04	0.07	0.04	0.03	0.07	0.05	0.05	0.05	0.04	0.05	0.04	0.07
	MDC	0.11	0.17	0.10	0.11	0.16	0.10	0.11	0.18	0.11	0.09	0.18	0.15	0.14	0.14	0.12	0.13	0.11	0.19
	CV	3.26	5.03	3.09	3.34	4.33	3.32	3.49	5.17	3.34	2.69	5.25	4.68	4.30	3.69	3.74	4.25	3.10	5.99

Abbreviations: C‐MYO, myotonometric creep; CV, coefficient of variation; D‐MYO, myotonometric decrement; F‐MYO, myotonometric frequency; ICC, intraclass correlation coefficient; LCI, lower 95% confidence interval; L, R, U, D, direction of measurement, left, right, up, down, respectively; MDC, minimal detectable change; R‐MYO, myotonometric relaxation time; SEM, standard error of the measurement; S‐MYO, myotonometric stiffness; UCI, upper 95% confidence interval; U1‐U3, D1‐D3, measurement points on the scar.

**TABLE 2:** Results of the inter‐rater reliability of the MyotonPRO parameter values in session 2.

Reliability statistical metrics		Measurement points on the scar																	
		U1			U2			U3			D1			D2			D3		
		L	U	R	L	U	R	L	U	R	L	D	R	L	D	R	L	D	R
F‐MYO	ICC	0.82	0.98	0.79	0.93	0.83	0.91	0.80	0.87	0.91	0.69	0.90	0.93	0.88	0.55	0.64	0.89	0.88	0.91
	LCI	0.66	0.95	0.62	0.86	0.68	0.82	0.63	0.76	0.83	0.46	0.80	0.86	0.77	0.26	0.38	0.79	0.78	0.83
	UCI	0.91	0.99	0.89	0.97	0.91	0.95	0.90	0.93	0.96	0.83	0.95	0.96	0.94	0.75	0.80	0.94	0.94	0.96
	SEM	0.64	0.47	0.49	0.40	1.10	0.46	0.62	1.03	0.36	0.79	0.84	0.34	0.54	1.79	0.81	0.59	0.81	0.44
	MDC	1.76	1.29	1.36	1.11	3.06	1.27	1.73	2.86	1.00	2.18	2.33	0.95	1.51	4.96	2.24	1.62	2.26	1.21
	CV	4.01	2.77	3.07	2.33	7.39	2.65	3.70	6.20	2.25	4.88	5.00	2.13	3.19	11.74	4.80	3.44	5.05	2.71
S‐MYO	ICC	0.77	0.95	0.75	0.86	0.94	0.91	0.90	0.90	0.93	0.83	0.97	0.94	0.88	0.88	0.73	0.90	0.90	0.94
	LCI	0.59	0.90	0.55	0.74	0.89	0.82	0.81	0.80	0.86	0.68	0.94	0.88	0.77	0.78	0.52	0.81	0.81	0.88
	UCI	0.88	0.98	0.87	0.93	0.97	0.95	0.95	0.95	0.97	0.91	0.98	0.97	0.94	0.94	0.85	0.95	0.95	0.97
	SEM	12.49	11.79	11.20	9.61	7.35	9.03	12.80	16.37	8.10	11.32	9.94	8.05	17.53	10.57	14.91	14.02	14.01	8.18
	MDC	34.62	32.68	31.06	26.63	20.36	25.04	35.48	45.38	22.46	31.38	27.56	22.31	48.60	29.31	41.32	38.87	38.84	22.66
	CV	5.64	4.51	4.68	3.77	3.36	3.43	4.98	6.21	3.42	4.92	3.98	3.30	7.70	4.20	5.84	5.31	5.64	3.50
D‐MYO	ICC	0.68	0.85	0.92	0.89	0.90	0.89	0.87	0.85	0.83	0.89	0.89	0.84	0.93	0.90	0.70	0.84	0.91	0.88
	LCI	0.45	0.71	0.85	0.79	0.80	0.79	0.76	0.72	0.69	0.79	0.79	0.71	0.86	0.81	0.48	0.71	0.83	0.78
	UCI	0.83	0.92	0.96	0.95	0.95	0.95	0.94	0.92	0.91	0.94	0.94	0.92	0.96	0.95	0.84	0.92	0.96	0.94
	SEM	0.14	0.12	0.05	0.08	0.14	0.10	0.09	0.14	0.12	0.07	0.13	0.09	0.09	0.10	0.15	0.10	0.10	0.09
	MDC	0.39	0.33	0.14	0.21	0.39	0.27	0.24	0.38	0.32	0.18	0.35	0.24	0.26	0.28	0.41	0.26	0.28	0.25
	CV	7.94	6.09	2.76	3.74	7.01	4.72	4.53	6.98	6.12	3.99	6.65	5.57	5.37	5.32	8.07	5.91	5.48	5.38
R‐MYO	ICC	0.78	0.96	0.84	0.93	0.95	0.93	0.91	0.89	0.91	0.88	0.96	0.93	0.93	0.87	0.80	0.92	0.93	0.92
	LCI	0.60	0.92	0.70	0.87	0.90	0.87	0.83	0.80	0.82	0.77	0.92	0.86	0.86	0.75	0.63	0.84	0.86	0.84
	UCI	0.88	0.97	0.91	0.96	0.97	0.96	0.95	0.95	0.95	0.94	0.98	0.96	0.96	0.93	0.89	0.96	0.96	0.96
	SEM	0.89	0.77	0.62	0.42	0.63	0.45	0.64	1.43	0.53	0.71	0.68	0.57	0.84	0.66	0.69	0.66	0.89	0.55
	MDC	2.45	2.13	1.73	1.17	1.73	1.24	1.77	3.97	1.47	1.96	1.89	1.58	2.32	1.84	1.92	1.83	2.46	1.53
	CV	4.19	3.97	3.08	2.20	2.76	2.39	3.36	7.28	2.62	3.48	3.36	2.90	3.80	3.50	3.68	3.59	4.36	2.75
C‐MYO	ICC	0.73	0.95	0.83	0.95	0.96	0.93	0.89	0.89	0.80	0.86	0.96	0.90	0.87	0.81	0.75	0.90	0.91	0.81
	LCI	0.52	0.90	0.68	0.90	0.93	0.86	0.79	0.79	0.63	0.74	0.91	0.80	0.76	0.65	0.55	0.81	0.83	0.66
	UCI	0.85	0.98	0.91	0.97	0.98	0.96	0.94	0.94	0.89	0.93	0.98	0.95	0.93	0.90	0.87	0.95	0.96	0.90
	SEM	0.05	0.05	0.03	0.02	0.03	0.02	0.04	0.08	0.04	0.04	0.04	0.04	0.07	0.04	0.04	0.04	0.05	0.04
	MDC	0.14	0.13	0.10	0.06	0.09	0.07	0.10	0.23	0.11	0.11	0.11	0.10	0.18	0.11	0.11	0.10	0.15	0.11
	CV	4.13	3.99	2.88	1.80	2.42	2.16	3.11	7.12	3.26	3.45	3.17	3.07	4.96	3.63	3.63	3.34	4.29	3.36

Abbreviations: C‐MYO, myotonometric creep; CV, coefficient of variation; D‐MYO, myotonometric decrement; F‐MYO, myotonometric frequency; ICC, intraclass correlation coefficient; LCI, lower 95% confidence interval; L, R, U, D, direction of measurement, left, right, up, down, respectively; MDC, minimal detectable change; R‐MYO, myotonometric relaxation time; SEM, standard error of the measurement; S‐MYO, myotonometric stiffness; UCI, upper 95% confidence interval; U1‐U3, D1‐D3, measurement points on the scar.

**TABLE 3:** Results of the intra‐rater reliability of the MyotonPRO parameter values for rater 1.

Reliability statistical metrics		Measurement points on the scar																	
		U1			U2			U3			D1			D2			D3		
		L	U	R	L	U	R	L	U	R	L	D	R	L	D	R	L	D	R
F‐MYO	ICC	0.88	0.94	0.79	0.69	0.79	0.67	0.76	0.80	0.83	0.63	0.85	0.56	0.64	0.22	0.59	0.73	0.68	0.61
	LCI	0.77	0.89	0.61	0.46	0.62	0.42	0.58	0.63	0.68	0.37	0.73	0.28	0.39	0.12	0.32	0.52	0.45	0.35
	UCI	0.94	0.97	0.89	0.83	0.89	0.82	0.88	0.90	0.91	0.80	0.93	0.75	0.81	0.52	0.78	0.86	0.83	0.79
	SEM	0.49	0.72	0.52	0.77	1.30	0.81	0.66	1.29	0.49	0.78	0.97	0.85	0.94	2.20	0.83	0.87	1.36	1.02
	MDC	1.35	1.99	1.44	2.14	3.60	2.24	1.84	3.57	1.35	2.17	2.69	2.36	2.60	6.11	2.29	2.41	3.77	2.82
	CV	3.07	4.36	3.21	4.55	8.77	4.73	4.00	8.01	3.02	4.89	5.96	5.30	5.62	14.93	4.98	5.22	8.63	6.28
S‐MYO	ICC	0.81	0.90	0.83	0.64	0.89	0.74	0.85	0.89	0.86	0.71	0.84	0.70	0.55	0.51	0.71	0.80	0.84	0.67
	LCI	0.65	0.81	0.68	0.39	0.78	0.54	0.71	0.78	0.74	0.49	0.70	0.47	0.26	0.21	0.49	0.63	0.70	0.43
	UCI	0.90	0.95	0.91	0.81	0.94	0.86	0.92	0.94	0.93	0.84	0.92	0.84	0.75	0.72	0.85	0.89	0.92	0.82
	SEM	10.80	16.17	10.99	15.39	10.07	15.63	15.33	17.54	12.05	12.36	20.67	18.27	21.99	33.49	15.44	18.68	18.87	20.44
	MDC	29.93	44.81	30.47	42.65	27.92	43.32	42.49	48.61	33.41	34.26	57.29	50.65	60.95	92.84	42.80	51.77	52.30	56.65
	CV	4.86	6.37	4.56	6.15	4.66	6.03	6.14	6.84	5.10	5.46	8.60	7.51	9.01	15.43	6.23	7.27	7.91	8.63
D‐MYO	ICC	0.80	0.84	0.74	0.71	0.66	0.79	0.74	0.75	0.53	0.58	0.75	0.42	0.61	0.65	0.54	0.54	0.79	0.65
	LCI	0.63	0.70	0.54	0.50	0.42	0.62	0.53	0.56	0.23	0.31	0.55	0.10	0.35	0.40	0.25	0.25	0.61	0.41
	UCI	0.89	0.92	0.86	0.85	0.82	0.89	0.86	0.87	0.73	0.77	0.87	0.67	0.79	0.81	0.74	0.74	0.89	0.81
	SEM	0.11	0.13	0.10	0.13	0.19	0.14	0.13	0.18	0.17	0.13	0.20	0.17	0.18	0.23	0.21	0.17	0.18	0.18
	MDC	0.31	0.36	0.28	0.36	0.53	0.39	0.35	0.51	0.48	0.37	0.56	0.47	0.51	0.63	0.58	0.48	0.50	0.49
	CV	6.26	6.59	5.54	6.32	9.85	6.85	6.81	9.24	9.21	8.23	10.57	11.11	10.04	13.70	11.40	10.90	9.90	10.12
R‐MYO	ICC	0.84	0.88	0.83	0.64	0.77	0.75	0.85	0.92	0.83	0.77	0.84	0.64	0.53	0.61	0.63	0.78	0.90	0.74
	LCI	0.71	0.77	0.69	0.38	0.59	0.56	0.72	0.84	0.69	0.58	0.69	0.39	0.24	0.34	0.37	0.61	0.81	0.54
	UCI	0.92	0.94	0.91	0.80	0.88	0.87	0.92	0.96	0.91	0.88	0.92	0.81	0.74	0.79	0.80	0.89	0.95	0.86
	SEM	0.70	1.39	0.67	0.97	1.32	0.87	0.81	1.35	0.71	0.90	1.36	1.14	1.25	1.81	0.92	1.02	1.02	1.03
	MDC	1.94	3.84	1.85	2.69	3.65	2.42	2.26	3.75	1.96	2.48	3.77	3.15	3.48	5.01	2.54	2.82	2.83	2.84
	CV	3.30	6.95	3.31	5.02	5.74	4.60	4.19	6.61	3.48	4.33	6.50	5.78	6.43	8.02	4.77	5.42	4.87	5.11
C‐MYO	ICC	0.81	0.87	0.80	0.64	0.73	0.72	0.80	0.91	0.74	0.74	0.76	0.57	0.45	0.63	0.53	0.69	0.83	0.69
	LCI	0.65	0.75	0.64	0.39	0.52	0.51	0.63	0.83	0.54	0.54	0.57	0.29	0.14	0.37	0.23	0.46	0.68	0.46
	UCI	0.90	0.93	0.90	0.81	0.85	0.85	0.89	0.95	0.86	0.86	0.87	0.76	0.68	0.80	0.73	0.83	0.91	0.83
	SEM	0.04	0.08	0.04	0.06	0.09	0.05	0.05	0.08	0.04	0.05	0.09	0.07	0.07	0.11	0.06	0.06	0.07	0.05
	MDC	0.11	0.23	0.11	0.15	0.25	0.14	0.13	0.22	0.12	0.15	0.24	0.19	0.18	0.29	0.15	0.16	0.20	0.15
	CV	3.12	6.98	3.20	4.73	6.50	4.36	4.06	6.54	3.50	4.31	6.84	5.81	5.77	8.00	4.90	5.31	5.87	4.58

Abbreviations: CV, coefficient of variation; C‐MYO, myotonometric creep; D‐MYO, myotonometric decrement; F‐MYO, myotonometric frequency; ICC, intraclass correlation coefficient; LCI, lower 95% confidence interval; L, R, U, D, direction of measurement, left, right, up, down, respectively; MDC, minimal detectable change; R‐MYO, myotonometric relaxation time; SEM, standard error of the measurement; S‐MYO, myotonometric stiffness; UCI, upper 95% confidence interval; U1‐U3, D1‐D3, measurement points on the scar.

**TABLE 4:** Results of the intra‐rater reliability of the MyotonPRO parameter values for rater 2.

Reliability statistical metrics		Measurement points on the scar																	
		U1			U2			U3			D1			D2			D3		
		L	U	R	L	U	R	L	U	R	L	D	R	L	D	R	L	D	R
F‐MYO	ICC	0.82	0.86	0.69	0.56	0.55	0.52	0.69	0.73	0.80	0.56	0.68	0.74	0.58	0.67	0.62	0.72	0.81	0.69
	LCI	0.66	0.74	0.46	0.27	0.26	0.22	0.46	0.52	0.63	0.28	0.45	0.53	0.30	0.43	0.35	0.51	0.66	0.45
	UCI	0.91	0.93	0.83	0.75	0.75	0.73	0.83	0.86	0.89	0.76	0.83	0.86	0.77	0.82	0.79	0.85	0.90	0.83
	SEM	0.60	1.04	0.60	1.03	1.84	1.06	0.78	1.53	0.56	0.90	1.52	0.66	1.05	1.38	0.89	0.96	1.10	0.83
	MDC	1.66	2.89	1.65	2.86	5.11	2.93	2.16	4.25	1.56	2.48	4.22	1.83	2.91	3.83	2.47	2.66	3.06	2.31
	CV	3.78	6.38	3.76	5.93	12.40	6.09	4.58	9.37	3.48	5.57	9.24	4.17	6.16	9.28	5.24	5.56	6.87	5.03
S‐MYO	ICC	0.78	0.87	0.69	0.74	0.83	0.66	0.91	0.82	0.85	0.52	0.76	0.77	0.66	0.68	0.56	0.77	0.86	0.72
	LCI	0.59	0.75	0.45	0.53	0.69	0.41	0.83	0.66	0.71	0.23	0.57	0.58	0.41	0.44	0.28	0.58	0.73	0.50
	UCI	0.88	0.93	0.83	0.86	0.91	0.81	0.95	0.90	0.92	0.73	0.87	0.88	0.81	0.83	0.76	0.88	0.93	0.85
	SEM	11.91	17.65	11.29	14.52	12.23	18.68	12.20	21.94	11.56	18.40	25.99	15.68	18.29	20.78	19.25	21.90	18.91	17.25
	MDC	33.01	48.94	31.30	40.24	33.90	51.79	33.80	60.80	32.03	51.00	72.04	43.47	50.71	57.59	53.36	60.70	52.41	47.81
	CV	5.36	6.95	4.79	5.63	5.72	7.05	4.59	8.45	4.82	8.09	10.80	6.57	7.22	9.51	7.37	8.19	7.61	7.13
D‐MYO	ICC	0.69	0.72	0.71	0.65	0.84	0.81	0.69	0.72	0.72	0.47	0.76	0.64	0.40	0.72	0.63	0.59	0.77	0.68
	LCI	0.45	0.51	0.49	0.40	0.70	0.65	0.47	0.51	0.50	0.16	0.56	0.38	0.08	0.51	0.38	0.32	0.59	0.45
	UCI	0.83	0.85	0.84	0.81	0.92	0.90	0.84	0.85	0.85	0.70	0.87	0.80	0.65	0.85	0.80	0.77	0.88	0.83
	SEM	0.13	0.17	0.11	0.15	0.14	0.13	0.15	0.19	0.14	0.18	0.20	0.14	0.22	0.20	0.17	0.16	0.17	0.16
	MDC	0.37	0.48	0.29	0.40	0.39	0.35	0.43	0.53	0.38	0.49	0.55	0.40	0.62	0.55	0.48	0.44	0.47	0.44
	CV	7.41	8.85	5.82	7.03	7.20	6.07	8.13	9.72	7.32	10.65	10.50	9.43	12.13	11.58	9.40	10.02	9.08	9.11
R‐MYO	ICC	0.82	0.85	0.76	0.73	0.82	0.71	0.89	0.78	0.88	0.59	0.66	0.72	0.65	0.47	0.61	0.74	0.87	0.67
	LCI	0.67	0.72	0.56	0.53	0.66	0.49	0.79	0.60	0.78	0.32	0.42	0.50	0.41	0.16	0.34	0.54	0.75	0.43
	UCI	0.91	0.92	0.87	0.86	0.90	0.84	0.94	0.89	0.94	0.77	0.82	0.85	0.81	0.70	0.79	0.86	0.93	0.82
	SEM	0.79	1.55	0.74	0.83	1.20	0.98	0.73	2.03	0.64	1.23	1.86	1.12	1.12	1.95	1.05	1.20	1.17	1.15
	MDC	2.19	4.30	2.05	2.29	3.34	2.71	2.02	5.61	1.77	3.40	5.15	3.11	3.10	5.41	2.90	3.34	3.25	3.18
	CV	3.75	7.64	3.62	4.38	5.24	5.25	3.88	10.09	3.17	6.00	8.99	5.63	5.91	8.69	5.65	6.66	5.75	5.86
C‐MYO	ICC	0.77	0.83	0.72	0.73	0.81	0.70	0.81	0.77	0.89	0.52	0.52	0.63	0.59	0.39	0.55	0.68	0.83	0.60
	LCI	0.58	0.68	0.51	0.52	0.66	0.48	0.65	0.58	0.78	0.23	0.23	0.37	0.31	0.06	0.26	0.44	0.68	0.33
	UCI	0.88	0.91	0.85	0.85	0.90	0.84	0.90	0.88	0.94	0.73	0.73	0.80	0.77	0.64	0.75	0.82	0.91	0.78
	SEM	0.05	0.09	0.04	0.05	0.08	0.05	0.05	0.12	0.03	0.07	0.12	0.07	0.07	0.13	0.06	0.07	0.07	0.06
	MDC	0.13	0.26	0.12	0.13	0.21	0.15	0.13	0.34	0.09	0.20	0.32	0.19	0.18	0.36	0.17	0.19	0.20	0.18
	CV	3.89	7.75	3.49	3.98	5.54	4.76	4.29	10.07	2.76	5.91	9.53	5.69	5.83	9.86	5.55	6.43	5.99	5.61

Abbreviations: CV, coefficient of variation; C‐MYO, myotonometric creep; D‐MYO, myotonometric decrement; F‐MYO, myotonometric frequency; ICC, intraclass correlation coefficient; LCI, lower 95% confidence interval; L, R, U, D, direction of measurement, left, right, up, down, respectively; MDC, minimal detectable change; R‐MYO, myotonometric relaxation time; SEM, standard error of the measurement; S‐MYO, myotonometric stiffness; UCI, upper 95% confidence interval; U1‐U3, D1‐D3, measurement points on the scar.

On both days, the testing procedure was the same and began by inviting the participant to lie supine on a treatment table and uncover her abdomen to expose the C‐section scar. A pillow was positioned under the participant's knees to minimise tension in the lower extremities and abdomen. R1 identified and marked six points on the skin adjacent to the scar (U1, U2 and U3, and D1, D2 and D3) using a waterproof pen. After a 5‐min relaxation period, R1 performed all 18 myotonometric measurements. Following another 5‐min break, R2 conducted the same 18 measurements. The following day, at approximately the same time, both raters (R1 and R2) repeated the measurements in the same manner. This procedure allowed us to estimate inter‐rater reliability (by comparing the results between the two raters for each session) and intra‐rater reliability (by comparing the results from the two testing sessions for each rater). During the testing procedure, both raters used the ‘Multiscan 5’ measurement mode, which involves taking five records per measurement. The rater placed the short arm of the L‐shaped MyotonPRO probe on the skin at the measurement point and attached it with a double‐sided adhesive washer. The rater then gently moved the myotonometer's body in the direction of the measurement until the green indicator light illuminated, activating the electromagnetic mechanism of the device (Figure 1C–F). This mechanism generated five successive mechanical impulses through the L‐shaped probe by applying a constant force to deform the skin horizontally at the measurement point. Although the myotonometer automatically produced a series of impulses, the rater held the device in place. Mechanical impulses caused oscillations in the scar skin, which were measured using an accelerometer built into the probe. The accelerometric data were then automatically processed by the MyotonPRO microprocessor to calculate five myotonometric parameters, as detailed in the following subsection.

### 2.4 Analysed Myotonometric (MYO) Parameters

The analysed MYO parameters included oscillation frequency (F‐MYO, [Hz]), stiffness (S‐MYO, [N/m]), logarithmic decrement of oscillations (D‐MYO, [log]), relaxation time (R‐MYO, [ms]) and creep (C‐MYO, [De, Deborah number]). According to previous studies [4, 27, 28], the interpretation criteria for myotonometric outcomes are as follows: higher F‐ and S‐MYO values indicate greater tissue tension and stiffness, whereas a higher D‐MYO value signifies greater dissipation of mechanical energy during oscillation and lower tissue elasticity. Conversely, lower values of R‐MYO and C‐MYO correspond to increased stiffness and tension, and reduced tissue elasticity [4, 27, 28].

### 2.5 Statistical Analysis

Demographic and anthropometric characteristics of the participants, including age, height, weight and BMI were assessed using descriptive statistics. Normality was assessed using the Kolmogorov–Smirnov test and Q‐Q plots.

The ICC_3,1_ (two‐way mixed model, single measures) with 95% confidence intervals (CIs) was used to examine the inter‐rater reliability (measurements performed on the same day by two raters) and intra‐rater reliability (measurements performed on two consecutive days by the same rater). Reliability was classified as excellent for ICC values greater than 0.9, good for values between 0.75 and 0.9, moderate for values between 0.5 and 0.74, and poor for values below 0.5 [29, 30].

Absolute reliability was assessed using the standard error of measurement (SEM), minimal detectable change (MDC) and coefficient of variation (CV). Bland–Altman plots were used to assess both inter‐rater and intra‐rater reliability, visualizing the degree of agreement and identifying any systematic bias.

A paired samples *t*‐test was used to compare the mean values of the myotonometric parameters between raters (inter‐rater comparisons) and sessions (intra‐rater comparisons). Data are presented as mean and standard deviation (SD). *p* ≤ 0.05 was considered statistically significant for all statistical analyses, which were performed using SPSS Statistics software (version 22.0; IBM, Armonk, NY, USA) and JASP 0.18.3 (University of Amsterdam, the Netherlands).

## 3 Results

### 3.1 Inter‐Rater and Intra‐Rater Reliability Based on ICC Values

**FIGURE 2:**
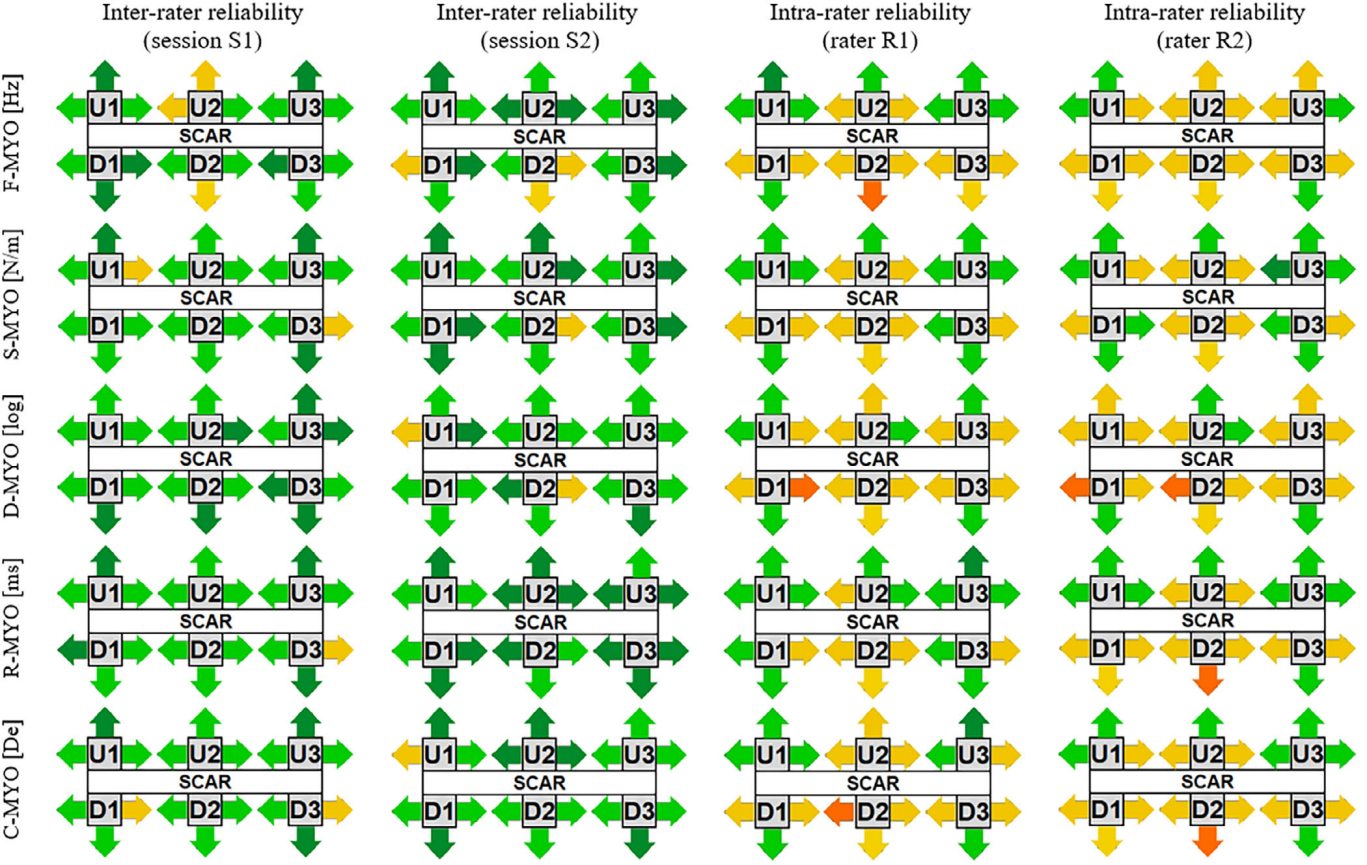
Graphical representation of ICC value ranges of the inter‐ and intra‐rater reliability agreement of the MyotonPRO parameter values, measurement points and measurement directions. F‐MYO, myotonometric frequency; S‐MYO, myotonometric stiffness; D‐MYO, myotonometric decrement; R‐MYO, myotonometric relaxation time; C‐MYO, myotonometric creep; U1–U3 represent measurement points up the scar, D1–D3 represent measurement points down the scar. Arrows indicate the directions of L‐shaped testing probe movement during myotonometric measurements, and their colours correspond to the ICC levels. Orange indicates poor reliability (ICC below 0.5); yellow, moderate reliability (ICC between 0.5 and 0.75); light green, good reliability (ICC between 0.75 and 0.9); and dark green, excellent reliability (ICC above 0.9).

The ICC values of the MYO parameters are presented in Figure 2 and Tables 1, 2, 3, 4. The schematic overview in Figure 2 illustrates the distribution of ICC categories across parameters (F‐MYO, S‐MYO, D‐MYO, R‐MYO, C‐MYO), measurement points (U1–U3, D1–D3) and measurement directions (down, up, left and right).

In both testing sessions, inter‐rater reliability was predominantly good to excellent. For example, in session 1, good reliability accounted for the majority of measurements across parameters, with additional values classified as excellent and only a small proportion moderate. In session 2, the proportion of excellent results increased, particularly for R‐MYO, where more than half of the measurements reached this level. No poor inter‐rater reliability was observed in either session.

Intra‐rater reliability showed a broader distribution. For rater R1, most values were moderate to good, with only occasional excellent or poor classifications. For rater R2, moderate reliability predominated, in some cases exceeding half of the measurements for a given parameter, while excellent reliability was rare. Instances of poor reliability occurred sporadically but never represented more than a small proportion of the results. Overall, inter‐rater reliability was higher than intra‐rater reliability, and no consistent effect of measurement point, direction or parameter was identified.

### 3.2 SEM, MDC and CV

Error and variability estimates are shown in Tables 1, 2, 3, 4. For the inter‐rater comparisons, SEM values ranged from 0.34–1.80 Hz for F‐MYO, 0.78–18.03 N/m for S‐MYO, 0.05–0.14 log for D‐MYO, 0.42–1.43 ms for R‐MYO and 0.02–0.07 De for C‐MYO. Intra‐rater SEM values were consistently higher, reaching 2.20 Hz for F‐MYO, 33.49 N/m for S‐MYO, 0.23 log for D‐MYO, 2.03 ms for R‐MYO and 0.13 De for C‐MYO. MDC values showed the same pattern: in inter‐rater comparisons, values were typically below 5 Hz for F‐MYO and 50 N/m for S‐MYO, whereas intra‐rater comparisons extended to more than 6 Hz and over 90 N/m, respectively. For the D‐MYO, R‐MYO and C‐MYO parameters, MDC values in inter‐rater analyses were modest (up to 0.41 log, 3.97 ms and 0.23 De, respectively), while intra‐rater analyses reached higher ranges (up to 0.63 log, 5.61 ms and 0.36 De, respectively).

The coefficients of variation also confirmed these trends. Inter‐rater CV values were relatively low, generally between 2% and 8% across parameters, while intra‐rater CVs were higher, in some cases exceeding 15% for F‐MYO and S‐MYO and 10% for C‐MYO. Together, these findings indicate narrower error margins and lower variability in inter‐rater than intra‐rater assessments.

### 3.3 Bland–Altman Analysis

**FIGURE 3:**
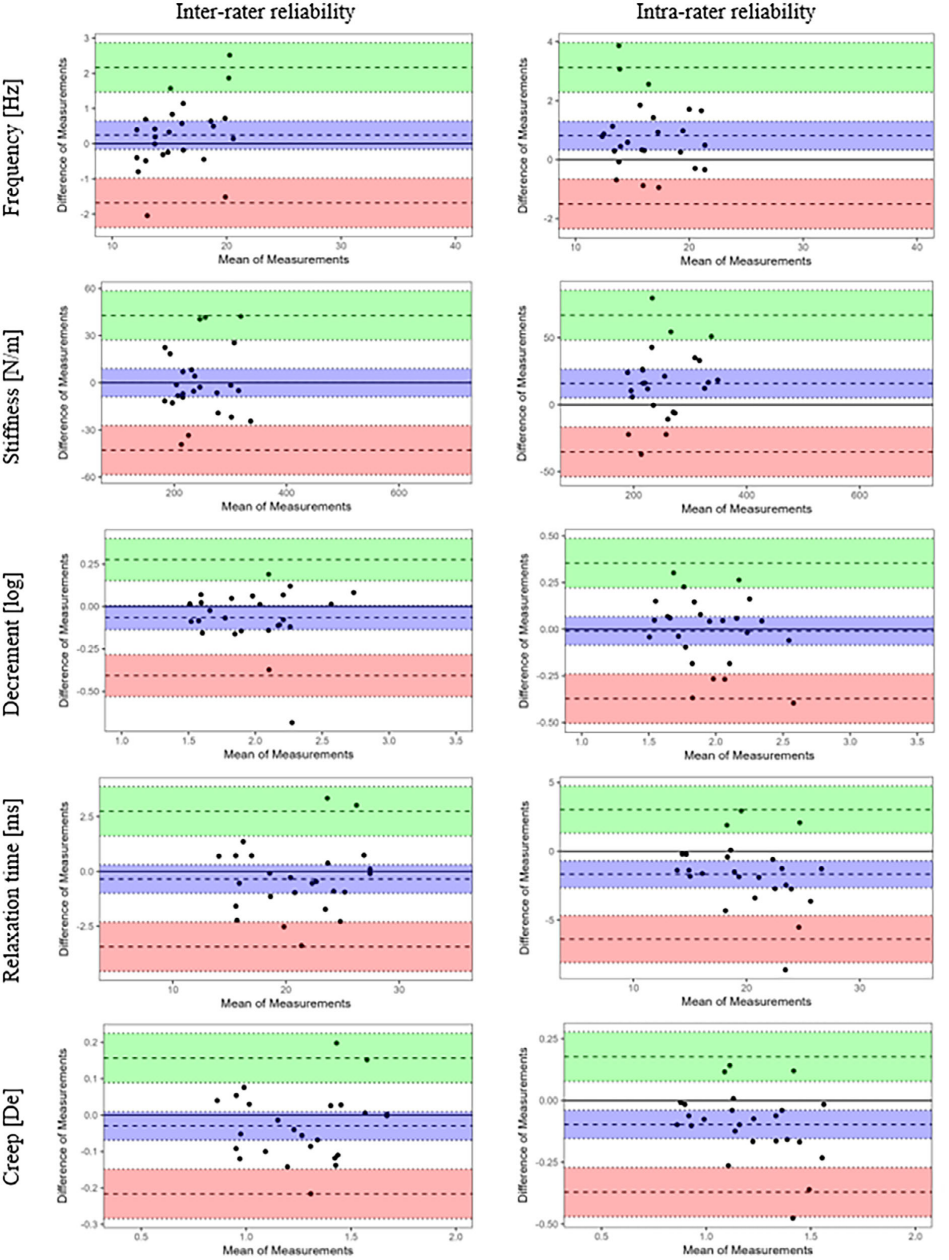
Representative Bland–Altman plots (including limits of agreement) of the inter‐rater and intra‐rater reliability agreement of MyotonPRO parameter values (point U1, measurement in the upward direction). The average parameter values obtained by the raters are shown on the X‐axis, and the differences between the raters/sessions are shown on the Y‐axis. The solid line indicates a zero value, the middle thick dotted line represents the mean difference, and the upper and lower thick dotted lines indicate the mean difference ± 1.96 standard deviation. Coloured areas indicate 95% confidence intervals.

Representative Bland–Altman plots are shown in Figure 3, with all plots included in the Supporting Information (Figure A.1–A.4). Numerical bias values and corresponding limits of agreement (LoA) ranges are provided in Tables A.1–A.4 of the Supporting Information. Inter‐rater analyses showed small biases close to zero, with differences between raters typically representing less than 5% of the mean values. Most data points were distributed symmetrically around the bias line and within the 95% LoA, with only occasional outliers that had minimal impact. Intra‐rater comparisons displayed larger biases, wider distributions around the bias line and broader LoA, with more frequent deviations beyond these limits, indicating higher variability between repeated measurements by the same rater across days.

### 3.4 Inter‐Rater and Intra‐Rater Comparisons of Myotonometric Parameters

Comparisons of parameter values between raters (Tables A.5–A.6) and between sessions (Tables A.7–A.8) revealed no statistically significant differences in most cases (*p* > 0.05). Where significant differences were found, the magnitude of change was always smaller than the corresponding MDC values, indicating that they were not clinically relevant.

## 4 Discussion

The present study aimed to evaluate the inter‐rater and intra‐rater reliability of the MyotonPRO device, equipped with an L‐shaped skin probe, for measuring the viscoelastic properties of C‐section scars. It should be noted that, due to its horizontal impulse application, the L‐shaped probe primarily assesses the mechanical properties of the superficial skin layers and adjacent scar tissue, while its sensitivity to alterations in deeper subcutaneous or myofascial structures is limited. As such, the present findings should be interpreted in the context of measurements reflecting predominantly surface‐level viscoelastic behaviour.

The findings showed that the MyotonPRO demonstrates good to excellent inter‐rater reliability and moderate to good intra‐rater reliability, supporting the hypothesis that the device is a reliable tool for this application. Across all metrics (ICC, CIs, SEM, MDC, CV and Bland–Altman plots), the inter‐rater reliability generally demonstrated less variability and greater precision than the intra‐rater reliability. This means that the measurements obtained within a single session by different raters were more consistent than those obtained by the same rater across day‐to‐day sessions, indicating that external factors or temporal changes may influence intra‐rater reliability.

### 4.1 Comparison of Inter‐Rater and Intra‐Rater Reliability Across Analysed Metrics

Interrater reliability was consistently strong, with ICC values frequently categorised as good to excellent and accompanied by narrow CIs, reflecting a high degree of agreement among the raters. These results indicate that measurements conducted within a short time interval by different raters remain stable, likely because of the controlled conditions enabled by the standardised use of the myotonometer's L‐shaped probe. In contrast, intra‐rater reliability typically showed lower ICC values, with many scores falling within the moderate range, indicating that repeated day‐to‐day assessments by the same rater introduce greater variability. Wider CIs in intra‐rater reliability further reflect this variability, suggesting that measurements are less stable across repeated sessions by the same individual.

Further insights into myotonometric measurement reliability were provided by the SEM, MDC and CV values. For inter‐rater measurements, lower SEM values suggested minimal error, reinforcing the precision of the assessments conducted by different raters. Similarly, the low MDC values observed in the inter‐rater reliability measurements imply that even small changes in tissue properties are detectable with relatively low error margins, underscoring the sensitivity of these measurements. Notably, in all cases where statistically significant inter‐rater differences were identified, their magnitude remained below the corresponding MDC thresholds, indicating that such differences are unlikely to reflect clinically meaningful changes (see Tables 1 and 2). By contrast, higher SEM and MDC values for intra‐rater measurements revealed a greater degree of fluctuation, indicating that repeated daily assessments by the same rater may produce slightly varying results across sessions.

Bland–Altman plots provide additional confirmation of these findings by revealing that inter‐rater measurements showed lower bias and tighter agreement compared to intra‐rater assessments. The distribution of Bland–Altman plots for inter‐rater reliability demonstrated reduced systematic differences, indicating that measurements between different raters align more closely when taken within a short time interval. This lack of bias highlights the ability of myotonometric assessments to produce consistent measurements when performed by various raters. This is advantageous for clinical use, where multiple practitioners may be involved. Conversely, Bland–Altman plots for intra‐rater reliability showed greater bias and variability, highlighting wider ranges of differences within the same rater's repeated measurements taken on two different days.

This suggests that intra‐rater reliability, when assessed over multiple days, is more susceptible to variations stemming from external factors or temporal changes, even under identical measurement conditions, rather than differences introduced by different raters within a single session. These fluctuations may result from minor inconsistencies in measurement techniques (e.g., slight variations in probe positioning) and/or subtle physiological changes in the scar and surrounding soft tissues due to time‐dependent factors such as patient fatigue and hydration status or environmental conditions like atmospheric pressure, both of which can influence measurement outcomes. Therefore, ensuring strict procedural consistency is crucial for enhancing the reliability of longitudinal assessments conducted by the same rater.

### 4.2 Comparison of Our Findings With Existing Literature

To the best of our knowledge, this is the first study to examine the inter‐rater and intra‐rater reliability of measuring the mechanical properties of C‐section scar skin using the MyotonPRO with the L‐shaped probe, employing various reliability indices such as ICC, CIs, SEM, MDC, CV and Bland–Altman plots. Therefore, we could not directly compare our findings with those of other studies. However, our findings align with those of Rosicka et al. [18, 19], who used the MyotonPRO device with the L‐shaped probe to assess the mechanical properties of unscarred skin, demonstrating high repeatability of such measurements.

Additionally, our results are consistent with those of previous studies demonstrating the reliability of the MyotonPRO device with the standard cylindrical probe for assessing the mechanical properties of various tissues [9].

Furthermore, our findings agree with those of Gilbert et al. [21], who employed the MyotonPRO with the standard cylindrical probe to assess the viscoelastic properties of both post‐caesarean section scar tissue and unscarred skin. Their study demonstrated good to excellent inter‐ and intra‐rater reliability of viscoelastic property measurements, with ICC values ranging from 0.99 to 1.00 and 0.87 to 0.98, respectively. Gilbert et al. [21] reported high reliability for measurements conducted perpendicularly using the standard cylindrical probe. However, our study specifically focused on the reliability of myotonometry with an L‐shaped probe for assessing post‐caesarean section scar tissue horizontally, similar to the pliability measurements used in the subjective assessment of scars with the pliability component of the POSAS. Thus, our findings and methodological approach contribute to the literature by addressing an aspect that has not yet been explored extensively.

### 4.3 Significance and Implications of the Findings

The high inter‐rater and reasonable intra‐rater reliabilities observed in this study provide a strong foundation for the incorporation of myotonometry into routine scar assessments. Using an L‐shaped probe enables clinicians to conduct reliable assessments of scar skin pliability that are less dependent on subjective interpretation, as seen in POSAS‐based assessments. Importantly, the absence of poor reliability scores and minimal clinically significant differences between the raters demonstrates that myotonometry provides a highly consistent method for scar assessment. This foundational reliability is crucial, as it enables clinicians to perform consistent evaluations over time, which is a necessary step to enhance the reproducibility of treatment monitoring. These findings have broad implications for scar assessment and management. The ability to reliably and noninvasively measure scar viscoelasticity opens up new possibilities for evaluating the effectiveness of various scar treatments, such as silicone gel sheets, laser therapy, or massage techniques, which are designed to improve scar pliability and reduce stiffness [20]. By providing quantifiable data, myotonometry enhances the precision of treatment monitoring, potentially leading to more tailored therapeutic approaches. Furthermore, minimal clinically significant differences between raters underscore the suitability of myotonometry in multi‐practitioner settings and larger research studies. Moderate to good intra‐rater reliability indicates that while the device performs well across different sessions, slight variability may occur, likely due to minor shifts in probe positioning, skin tension, or natural changes in scar properties. Standardised protocols can further mitigate intra‐rater variability and improve the accuracy of long‐term monitoring.

### 4.4 Limitations

Despite these promising results, this study had several limitations. First, the sample size was relatively small (*n* = 25). Although this number is sufficient for reliability testing, larger studies are needed to confirm these findings and explore the potential variability in different populations, such as those with hypertrophic or keloid scars. Additionally, the study was limited to scars at a specific healing stage (6–7 weeks post‐caesarean section), which may not fully represent the range of mechanical properties that scars exhibit at different stages of healing. Another limitation is the focus on the L‐shaped probe, which, although designed specifically for skin measurements, may not capture the full depth of the mechanical changes that occur in deeper scar tissue layers. Future studies could compare the L‐shaped probe with a standard probe to determine whether different probe designs offer complementary information regarding scar tissue. Moreover, although the POSAS scale is a relevant comparator for our outcomes, correlations with MYO parameters were not included in the present study to maintain a clear and focused scope on myotonometry reliability, given the large dataset and number of analyses.

### 4.5 Future Research Directions

Future research should aim to include a more diverse population than the one examined in this study, which was limited to completely healed C‐section scars at a specific healing stage. A more diverse population would encompass individuals with various types of scars (e.g., hypertrophic or keloid scars) and scars at different stages of maturation to determine myotonometry's reliability in a broader clinical context.

Moreover, longitudinal studies are needed to provide insights into how the mechanical properties of scars change over time and to correlate these changes with clinical outcomes, which would enhance the reproducibility of treatment monitoring. To fully evaluate the objectivity of myotonometry, future studies should compare its outcomes with those obtained from cutometry [31], elastography [32] and other available methods that demonstrate comparable repeatability [33, 34, 35]. Establishing this convergent validity is a crucial step before correlating MYO data with widely used clinical assessments, such as visual scar scales and patient‐reported outcomes (e.g., POSAS), to confirm its clinical relevance.

Finally, given the observed influence of temporal factors on intra‐rater reliability, future studies should investigate whether shorter time intervals between sessions (e.g., within the same day) can improve intra‐rater measurement stability. The impact of external factors such as time of day, participant hydration and fatigue and atmospheric conditions should also be specifically examined to enhance the precision of assessments.

## 5 Conclusions

In conclusion, this study demonstrates that the MyotonPRO device, with an L‐shaped skin probe, provides good to excellent inter‐rater reliability and moderate to good intra‐rater reliability for assessing the viscoelastic properties of C‐section scar skin. These findings support the use of myotonometry as a reliable tool for scar assessment with potential applications in both clinical practice and research.

## Conflicts of Interest

The authors declare that there is no conflict of interest regarding the publication of this article. All authors confirm that they have no financial or personal relationships with other people or organizations that could inappropriately influence (bias) their work.

## Supporting information



Table A.1. Numerical bias values and corresponding 95% limits of agreement for inter‐rater measurements obtained during session S1.

Table A.2. Numerical bias values and corresponding 95% limits of agreement for inter‐rater measurements obtained during session S2.

Table A.3. Numerical bias values and corresponding 95% limits of agreement for intra‐rater measurements obtained by rater R1.

Table A.4. Numerical bias values and corresponding 95% limits of agreement for intra‐rater measurements obtained by rater R2.

Table A.5. Inter‐rater comparison of MyotonPRO parameter values recorded during measurement session S1

Table A.6. Inter‐rater comparison of MyotonPRO parameter values recorded during measurement session S2.

Table A.7. Comparison of MyotonPRO parameter values across sessions performed by rater R1

Table A.8. Comparison of MyotonPRO parameter values across sessions performed by rater R2.

## Data Availability

The data that support the findings of this study are openly available in Zenodo at https://zenodo.org/uploads/15193385.
