# Supplementary material for: Inter‐ and Intra‐Rater Reliability of Myotonometric Assessment of the Mechanical Properties of Caesarean Section Scar Skin Using the MyotonPRO With an L‐Shaped Probe
Source: Skin Res Technol. 2026 Jan 9;32(1):e70315. doi: 10.1111/srt.70315 (PMC12784373; doi:10.1111/srt.70315)
Supplement: Supplementary file 1 — Table A.1. Numerical bias values and corresponding 95% limits of agreement for inter‐rater measurements obtained during session S1. [file SRT-32-e70315-s003.pdf]

Table A.1. Numerical bias values and corresponding 95% limits of agreement for inter-rater measurements obtained during session S1.

		**Bland-Altman metrics**	**F-MYO**			**S-MYO**			**D-MYO**			**R-MYO**			**C-MYO**		
			Estimate	Lower CI	Upper CI	Estimate	Lower CI	Upper CI	Estimate	Lower CI	Upper CI	Estimate	Lower CI	Upper CI	Estimate	Lower CI	Upper CI
U1	L	Mean Bias	-0.018	-0.324	0.288	-1.72	-8.29	4.85	-0.047	-0.104	0.010	0.278	-0.154	0.709	0.013	-0.012	0.039
		Lower Limit of Agreement	-1.469	-1.815	-1.124	-32.89	-40.31	-25.48	-0.318	-0.383	-0.254	-1.770	-2.257	-1.283	-0.107	-0.135	-0.078
		Upper Limit of Agreement	1.433	1.088	1.779	29.45	22.04	36.87	0.224	0.159	0.288	2.325	1.838	2.812	0.133	0.105	0.162
	U	Mean Bias	0.245	-0.160	0.650	0.02	-9.00	09.04	-0.065	-0.137	0.006	-0.342	-0.993	0.308	-0.030	-0.069	0.010
		Lower Limit of Agreement	-1.678	-2.136	-1.221	-42.81	-52.99	-32.62	-0.407	-0.488	-0.326	-3.431	-4.165	-2.696	-0.216	-0.261	-0.172
		Upper Limit of Agreement	2.169	1.711	2.626	42.85	32.67	53.04	0.276	0.195	0.357	2.746	2.011	3.480	0.157	0.113	0.201
	R	Mean Bias	0.694	-0.056	1.440	11.00	-2.65	24.60	0.023	-0.033	0.080	-0.454	-0.959	0.051	-0.030	-0.057	-0.004
		Lower Limit of Agreement	-2.867	-3.714	-2.020	-53.70	-69.02	-38.30	-0.243	-0.307	-0.180	-2.852	-3.423	-2.282	-0.157	-0.187	-0.127
		Upper Limit of Agreement	4.254	3.407	5.100	75.60	60.19	90.90	0.290	0.227	0.353	1.943	1.373	2.514	0.096	0.066	0.127
U2	L	Mean Bias	-0.817	-1.420	-0.211	-12.50	-25.00	-0.05	-0.062	-0.118	-0.005	0.701	0.229	1.170	0.037	0.009	0.066
		Lower Limit of Agreement	-3.695	-4.380	-3.010	-71.70	-85.80	-57.63	-0.330	-0.394	-0.267	-1.541	-2.074	-1.010	-0.099	-0.131	-0.066
		Upper Limit of Agreement	2.061	1.380	2.746	46.70	32.60	60.76	0.207	0.143	0.271	2.942	2.409	3.480	0.173	0.141	0.206
	U	Mean Bias	-0.826	-1.920	0.267	-0.78	-6.95	5.40	-0.105	-0.178	-0.032	0.165	-0.419	0.749	0.009	-0.029	0.046
		Lower Limit of Agreement	-6.016	-7.250	-4.781	-30.10	-37.08	-23.13	-0.451	-0.533	-0.368	-2.608	-3.268	-1.948	-0.169	-0.211	-0.127
		Upper Limit of Agreement	4.364	3.130	5.599	28.55	21.58	35.53	0.241	0.159	0.324	2.938	2.278	3.597	0.186	0.144	0.229
	R	Mean Bias	-0.195	-0.558	0.168	-3.52	-10.70	3.64	-0.016	-0.069	0.037	0.265	-0.096	0.625	0.007	-0.015	0.029
		Lower Limit of Agreement	-1.918	-2.328	-1.508	-37.50	-45.60	-29.42	-0.267	-0.327	-0.208	-1.446	-1.853	-1.039	-0.098	-0.123	-0.073
		Upper Limit of Agreement	1.529	1.119	1.939	30.46	22.40	38.54	0.236	0.176	0.296	1.975	1.569	2.382	0.112	0.087	0.137
U3	L	Mean Bias	-0.526	-0.888	-0.163	-16.00	-24.90	-7.15	-0.040	-0.091	0.011	0.820	0.402	1.238	0.034	0.011	0.058
		Lower Limit of Agreement	-2.248	-2.658	-1.838	-58.20	-68.20	-48.17	-0.282	-0.340	-0.225	-1.164	-1.636	-0.692	-0.078	-0.104	-0.051
		Upper Limit of Agreement	1.197	0.787	1.606	26.10	16.10	36.16	0.202	0.145	0.260	2.804	2.332	3.276	0.146	0.119	0.173
	U	Mean Bias	-0.301	-0.727	0.125	-5.39	-13.10	2.35	-0.003	-0.059	0.054	0.466	-0.440	1.370	0.023	-0.032	0.078
		Lower Limit of Agreement	-2.325	-2.806	-1.843	-42.15	-50.90	-33.41	-0.271	-0.335	-0.207	-3.833	-4.855	-2.810	-0.237	-0.298	-0.175
		Upper Limit of Agreement	1.723	1.242	2.204	31.37	22.60	40.11	0.266	0.202	0.330	4.764	3.741	5.790	0.283	0.221	0.345
	R	Mean Bias	-0.288	-0.656	0.080	-6.09	-14.50	2.30	0.022	-0.037	0.081	0.284	-0.162	0.730	0.004	-0.023	0.031
		Lower Limit of Agreement	-2.035	-2.451	-1.620	-45.91	-55.40	-36.43	-0.258	-0.324	-0.191	-1.832	-2.335	-1.329	-0.124	-0.154	-0.093
		Upper Limit of Agreement	1.459	1.044	1.875	33.73	24.30	43.20	0.301	0.235	0.368	2.400	1.897	2.903	0.132	0.102	0.163

		Mean Bias	-0.126	-0.425	0.173	-0.88	-7.25	5.49	-0.022	-0.058	0.013	0.365	-0.225	0.954	0.026	-0.014	0.065
	L	Lower Limit of Agreement	-1.547	-1.886	-1.209	-31.11	-38.30	-23.92	-0.190	-0.230	-0.150	-2.434	-3.100	-1.768	-0.161	-0.205	-0.117
		Upper Limit of Agreement	1.295	0.957	1.634	29.35	22.16	36.54	0.145	0.105	0.185	3.164	2.498	3.830	0.212	0.168	0.257
		Mean Bias	-0.032	-0.321	0.257	-2.32	-9.46	4.82	-0.026	-0.100	0.048	0.094	-0.222	0.410	0.001	-0.019	0.021
D1	D	Lower Limit of Agreement	-1.406	-1.732	-1.079	-36.20	-44.26	-28.14	-0.377	-0.460	-0.293	-1.407	-1.763	-1.050	-0.094	-0.117	-0.072
		Upper Limit of Agreement	1.342	1.015	1.668	31.56	23.50	39.62	0.325	0.241	0.408	1.594	1.237	1.950	0.096	0.073	0.118
		Mean Bias	0.228	-0.020	0.475	1.27	-7.07	9.62	0.038	-0.034	0.109	-0.322	-0.728	0.085	-0.024	-0.055	0.008
	R	Lower Limit of Agreement	-0.946	-1.225	-0.667	-38.35	-47.77	-28.92	-0.300	-0.380	-0.220	-2.250	-2.708	-1.791	-0.174	-0.209	-0.138
		Upper Limit of Agreement	1.401	1.122	1.680	40.89	31.46	50.31	0.375	0.295	0.455	1.606	1.148	2.065	0.126	0.091	0.162
		Mean Bias	-0.231	-0.936	0.474	-7.30	-16.70	02.05	-0.076	-0.147	-0.005	0.542	-0.012	1.100	0.026	-0.005	0.057
	L	Lower Limit of Agreement	-3.579	-4.375	-2.783	-51.70	-62.30	-41.14	-0.414	-0.494	-0.333	-2.089	-2.715	-1.460	-0.123	-0.158	-0.087
		Upper Limit of Agreement	3.117	2.320	3.913	37.09	26.50	47.65	0.262	0.182	0.342	3.174	2.548	3.800	0.175	0.139	0.210
		Mean Bias	-0.787	-1.490	-0.080	-16.00	-33.80	1.71	-0.010	-0.074	0.054	0.620	0.098	1.140	0.027	-0.003	0.058
D2	D	Lower Limit of Agreement	-4.144	-4.940	-3.346	-100.30	-120.40	-80.28	-0.313	-0.385	-0.241	-1.859	-2.449	-1.270	-0.116	-0.150	-0.082
		Upper Limit of Agreement	2.571	1.770	3.369	68.30	48.20	88.31	0.294	0.222	0.366	3.099	2.509	3.690	0.171	0.137	0.205
		Mean Bias	-0.599	-1.050	-0.143	-16.50	-32.40	-0.51	0.053	-0.035	0.140	0.583	0.192	0.974	0.023	-0.002	0.049
	R	Lower Limit of Agreement	-2.761	-3.280	-2.247	-92.20	-110.30	-74.22	-0.365	-0.464	-0.266	-1.272	-1.714	-0.831	-0.098	-0.127	-0.069
		Upper Limit of Agreement	1.563	1.050	2.078	59.30	41.30	77.33	0.470	0.371	0.569	2.439	1.997	2.880	0.145	0.116	0.174
		Mean Bias	-0.389	-1.020	0.246	-9.12	-18.00	-0.21	-0.053	-0.128	0.022	0.586	0.080	1.090	0.024	-0.004	0.052
	L	Lower Limit of Agreement	-3.407	-4.120	-2.689	-51.42	-61.50	-41.36	-0.409	-0.494	-0.324	-1.819	-2.391	-1.250	-0.110	-0.142	-0.078
		Upper Limit of Agreement	2.628	1.910	3.340	33.18	23.10	43.24	0.304	0.219	0.388	2.991	2.419	3.560	0.159	0.126	0.190
		Mean Bias	-0.626	-1.031	-0.221	-15.30	-27.50	-3.02	0.004	-0.050	0.058	0.850	0.398	1.303	0.044	0.017	0.071
D3	D	Lower Limit of Agreement	-2.550	-3.007	-2.092	-73.40	-87.20	-59.58	-0.254	-0.315	-0.192	-1.300	-1.811	-0.788	-0.085	-0.116	-0.054
		Upper Limit of Agreement	1.298	0.840	1.755	42.90	29.00	56.71	0.262	0.201	0.323	3.000	2.489	3.512	0.173	0.142	0.204
		Mean Bias	-0.498	-1.020	0.026	-7.70	-22.40	6.99	0.048	-0.034	0.129	0.584	-0.062	1.230	0.029	-0.012	0.071
	R	Lower Limit of Agreement	-2.989	-3.580	-2.397	-77.45	-94.00	-60.86	-0.341	-0.434	-0.249	-2.485	-3.216	-1.760	-0.166	-0.212	-0.119
		Upper Limit of Agreement	1.992	1.400	2.585	62.04	45.50	78.64	0.436	0.344	0.529	3.653	2.923	4.380	0.225	0.178	0.271

U1-U3, D1-D3, measurement points on the scar; L, R, U, D, direction of measurement, left, right, up, down, respectively; F-MYO, myotonometric frequency, S-MYO, myotonometric stiffness, D-MYO, myotonometric decrement, R-MYO, myotonometric relaxation time, C-MYO, myotonometric creep; CI, confidence interval.
