# Supplementary material for: Inter‐ and Intra‐Rater Reliability of Myotonometric Assessment of the Mechanical Properties of Caesarean Section Scar Skin Using the MyotonPRO With an L‐Shaped Probe
Source: Skin Res Technol. 2026 Jan 9;32(1):e70315. doi: 10.1111/srt.70315 (PMC12784373; doi:10.1111/srt.70315)
Supplement: Supplementary file 2 — Table A.2. Numerical bias values and corresponding 95% limits of agreement for inter‐rater measurements obtained during session S2. [file SRT-32-e70315-s007.pdf]

Table A.2. Numerical bias values and corresponding 95% limits of agreement for inter-rater measurements obtained during session S2.

		Bland-Altman metrics	F-MYO			S-MYO			D-MYO			R-MYO			C-MYO		
			Estimate	Lower CI	Upper CI	Estimate	Lower CI	Upper CI	Estimate	Lower CI	Upper CI	Estimate	Lower CI	Upper CI	Estimate	Lower CI	Upper CI
U1	L	Mean Bias	0.215	-0.259	0.688	3.18	-6.26	12.60	-0.011	-0.094	0.072	-0.275	-0.820	0.270	-0.021	-0.052	0.010
		Lower Limit of Agreement	-2.034	-2.569	-1.499	-41.66	-52.33	-31.00	-0.404	-0.498	-0.311	-2.862	-3.477	-2.247	-0.168	-0.203	-0.133
		Upper Limit of Agreement	2.463	1.928	2.998	48.03	37.36	58.70	0.382	0.289	0.476	2.312	1.696	2.927	0.126	0.091	0.161
	U	Mean Bias	0.033	-0.269	0.335	2.78	-4.61	10.20	-0.015	-0.083	0.054	0.182	-0.281	0.646	0.017	-0.011	0.045
		Lower Limit of Agreement	-1.400	-1.740	-1.059	-32.28	-40.62	-23.90	-0.341	-0.419	-0.264	-2.017	-2.540	-1.494	-0.116	-0.148	-0.084
		Upper Limit of Agreement	1.466	1.125	1.807	37.83	29.49	46.20	0.312	0.235	0.390	2.382	1.859	2.905	0.150	0.118	0.182
	R	Mean Bias	-0.016	-0.490	0.458	0.65	-13.20	14.50	-6.40e−4	-0.038	0.037	0.254	-0.178	0.686	0.012	-0.012	0.036
		Lower Limit of Agreement	-2.266	-2.801	-1.730	-65.09	-80.70	-49.50	-0.179	-0.221	-0.137	-1.797	-2.285	-1.309	-0.103	-0.130	-0.076
		Upper Limit of Agreement	2.234	1.698	2.769	66.39	50.70	82.00	0.178	0.135	0.220	2.305	1.817	2.793	0.127	0.099	0.154
U2	L	Mean Bias	0.592	-0.132	1.320	15.20	-1.01	31.40	0.017	-0.040	0.074	-0.612	-0.968	-0.256	-0.031	-0.051	-0.011
		Lower Limit of Agreement	-2.848	-3.666	-2.030	-6 1.70	-80.01	-43.40	-0.252	-0.316	-0.188	-2.300	-2.702	-1.899	-0.125	-0.147	-0.102
		Upper Limit of Agreement	4.032	3.214	4.850	92.10	73.81	110.40	0.286	0.222	0.350	1.076	0.675	1.478	0.063	0.040	0.085
	U	Mean Bias	-0.520	-1.160	0.117	-4.62	-9.19	-0.05	-0.066	-0.146	0.013	0.342	-0.054	0.739	0.008	-0.013	0.029
		Lower Limit of Agreement	-3.545	-4.270	-2.826	-26.31	-31.47	-21.15	-0.444	-0.534	-0.354	-1.540	-1.988	-1.092	-0.093	-0.118	-0.069
		Upper Limit of Agreement	2.506	1.790	3.225	17.08	11.92	22.24	0.311	0.222	0.401	2.225	1.777	2.673	0.109	0.085	0.133
	R	Mean Bias	0.419	0.057	0.781	9.78	0.32	19.20	0.025	-0.036	0.086	-0.309	-0.579	-0.038	-0.015	-0.029	-1.77e−5
		Lower Limit of Agreement	-1.300	-1.709	-0.891	-35.15	-45.84	-24.50	-0.264	-0.333	-0.195	-1.593	-1.898	-1.287	-0.085	-0.101	-0.068
		Upper Limit of Agreement	2.138	1.729	2.547	54.72	44.03	65.40	0.314	0.245	0.382	0.975	0.670	1.281	0.055	0.039	0.072
U3	L	Mean Bias	0.567	0.063	1.070	14.90	3.64	26.10	0.046	-0.010	0.101	-0.629	-1.027	-0.231	-0.033	-0.056	-0.011
		Lower Limit of Agreement	-1.823	-2.392	-1.250	-38.50	-51.15	-25.80	-0.217	-0.280	-0.155	-2.520	-2.969	-2.070	-0.140	-0.166	-0.115
		Upper Limit of Agreement	2.957	2.388	3.000	68.20	55.52	80.90	0.309	0.246	0.371	1.262	0.812	1.712	0.074	0.049	0.100
	U	Mean Bias	0.495	-0.156	1.150	3.28	-6.58	13.10	-0.076	-0.154	0.003	-0.843	-1.740	0.049	-0.058	-0.111	-0.004
		Lower Limit of Agreement	-2.595	-3.330	-1.860	-43.52	-54.66	-32.40	-0.449	-0.538	-0.360	-5.081	-6.090	-4.073	-0.313	-0.374	-0.252
		Upper Limit of Agreement	3.584	2.849	4.320	50.08	38.95	61.20	0.298	0.209	0.387	3.394	2.390	4.402	0.198	0.137	0.259
	R	Mean Bias	0.251	-0.192	0.693	7.98	-2.84	18.80	0.023	-0.064	0.110	-0.265	-0.683	0.153	-0.007	-0.034	0.020
		Lower Limit of Agreement	-1.850	-2.350	-1.350	-43.36	-55.58	-31.20	-0.389	-0.487	-0.291	-2.250	-2.723	-1.778	-0.135	-0.165	-0.104
		Upper Limit of Agreement	2.351	1.852	2.851	59.32	47.10	71.50	0.435	0.337	0.533	1.721	1.248	2.193	0.121	0.091	0.152

		Mean Bias	0.262	-0.200	0.724	2.56	-4.07	9.19	-8.80e−4	-0.045	0.043	-0.339	-0.744	0.065	-0.025	-0.048	-0.001
	L	Lower Limit of Agreement	-1.933	-2.455	-1.411	-28.90	-36.38	-21.42	-0.208	-0.258	-0.159	-2.259	-2.716	-1.803	-0.137	-0.164	-0.110
		Upper Limit of Agreement	2.457	1.935	2.979	34.02	26.54	41.50	0.207	0.157	0.256	1.581	1.124	2.038	0.087	0.061	0.114
		Mean Bias	0.589	-0.292	1.470	8.34	-8.99	25.70	-0.011	-0.100	0.078	-0.488	-1.170	0.195	-0.026	-0.063	0.011
D1	D	Lower Limit of Agreement	-3.593	-4.588	-2.600	-73.94	-93.51	-54.40	-0.432	-0.532	-0.332	-3.733	-4.500	-2.961	-0.202	-0.244	-0.160
		Upper Limit of Agreement	4.770	3.776	5.770	90.61	71.04	110.20	0.409	0.309	0.510	2.757	1.980	3.529	0.150	0.108	0.192
		Mean Bias	-0.330	-0.681	0.020	-7.88	-15.20	-0.53	0.002	-0.050	0.054	0.304	-0.040	0.648	0.014	-0.008	0.035
	R	Lower Limit of Agreement	-1.995	-2.390	-1.599	-42.80	-51.10	-34.49	-0.246	-0.305	-0.187	-1.328	-1.716	-0.939	-0.090	-0.115	-0.066
		Upper Limit of Agreement	1.334	0.938	1.730	27.04	18.70	35.34	0.250	0.191	0.309	1.936	1.548	2.324	0.117	0.093	0.142
		Mean Bias	0.105	-0.280	0.490	8.18	0.51	15.90	0.032	-0.028	0.092	-0.562	-0.961	-0.164	-0.022	-0.047	0.002
	L	Lower Limit of Agreement	-1.722	-2.157	-1.288	-28.25	-36.92	-19.60	-0.254	-0.322	-0.186	-2.454	-2.904	-2.004	-0.139	-0.167	-0.111
		Upper Limit of Agreement	1.932	1.497	2.367	44.62	35.95	53.30	0.318	0.250	0.386	1.329	0.879	1.779	0.095	0.067	0.122
		Mean Bias	0.233	-0.827	1.290	0.86	-10.30	12.00	0.021	-0.033	0.076	0.126	-0.385	0.637	0.012	-0.027	0.051
D2	D	Lower Limit of Agreement	-4.800	-5.998	-3.600	-52.11	-64.70	-39.50	-0.237	-0.298	-0.176	-2.301	-2.878	-1.724	-0.172	-0.216	-0.129
		Upper Limit of Agreement	5.266	4.069	6.460	53.82	41.20	66.40	0.279	0.218	0.341	2.552	1.975	3.129	0.197	0.153	0.241
		Mean Bias	0.136	-0.517	0.789	13.80	3.34	24.20	0.077	-0.009	0.162	-0.754	-1.184	-0.323	-0.031	-0.057	-0.006
	R	Lower Limit of Agreement	-2.966	-3.703	-2.228	-35.80	-47.58	-24.00	-0.329	-0.425	-0.232	-2.798	-3.285	-2.312	-0.153	-0.182	-0.124
		Upper Limit of Agreement	3.237	2.499	3.975	63.30	51.55	75.10	0.482	0.386	0.579	1.291	0.805	1.777	0.090	0.061	0.119
		Mean Bias	0.626	0.288	0.964	10.70	2.49	18.80	-0.001	-0.057	0.055	-0.586	-0.964	-0.207	-0.030	-0.051	-0.009
	L	Lower Limit of Agreement	-0.980	-1.362	-0.598	-28.10	-37.36	-18.90	-0.267	-0.331	-0.204	-2.382	-2.809	-1.954	-0.130	-0.154	-0.107
		Upper Limit of Agreement	2.231	1.849	2.613	49.50	40.23	58.70	0.266	0.202	0.329	1.211	0.783	1.638	0.070	0.046	0.093
		Mean Bias	0.450	-0.016	0.915	14.20	6.24	22.30	0.043	-0.019	0.103	-0.730	-1.240	-0.223	-0.037	-0.067	-0.007
D3	D	Lower Limit of Agreement	-1.760	-2.286	-1.234	-23.80	-32.84	-14.70	-0.247	-0.316	-0.178	-3.137	-3.710	-2.565	-0.180	-0.214	-0.146
		Upper Limit of Agreement	2.660	2.134	3.186	52.30	43.24	61.30	0.332	0.263	0.401	1.678	1.110	2.251	0.106	0.072	0.140
		Mean Bias	0.605	0.205	1.005	9.79	2.53	17.10	0.003	-0.052	0.057	-0.551	-0.886	-0.217	-0.022	-0.045	0.002
	R	Lower Limit of Agreement	-1.294	-1.746	-0.842	-24.68	-32.88	-16.50	-0.255	-0.317	-0.194	-2.140	-2.517	-1.762	-0.134	-0.161	-0.107
		Upper Limit of Agreement	2.505	2.053	2.957	44.26	36.06	52.50	0.261	0.199	0.322	1.037	0.659	1.415	0.091	0.064	0.117

U1-U3, D1-D3, measurement points on the scar; L, R, U, D, direction of measurement, left, right, up, down, respectively; F-MYO, myotonometric frequency, S-MYO, myotonometric stiffness, D-MYO, myotonometric decrement, R-MYO, myotonometric relaxation time, C-MYO, myotonometric creep; CI, confidence interval.
