# Supplementary material for: Inter‐ and Intra‐Rater Reliability of Myotonometric Assessment of the Mechanical Properties of Caesarean Section Scar Skin Using the MyotonPRO With an L‐Shaped Probe
Source: Skin Res Technol. 2026 Jan 9;32(1):e70315. doi: 10.1111/srt.70315 (PMC12784373; doi:10.1111/srt.70315)
Supplement: Supplementary file 3 — Table A.3. Numerical bias values and corresponding 95% limits of agreement for intra‐rater measurements obtained by rater R1. [file SRT-32-e70315-s008.pdf]

Table A.3. Numerical bias values and corresponding 95% limits of agreement for intra-rater measurements obtained by rater R1.

		**Bland-Altman metrics**	**F-MYO**			**S-MYO**			**D-MYO**			**R-MYO**			**C-MYO**		
			Estimate	Lower CI	Upper CI	Estimate	Lower CI	Upper CI	Estimate	Lower CI	Upper CI	Estimate	Lower CI	Upper CI	Estimate	Lower CI	Upper CI
U1	L	Mean Bias	-0.094	-0.430	0.242	-2.34	-12.10	7.38	0.015	-0.058	0.088	0.189	-0.332	0.710	0.007	-0.021	0.035
		Lower Limit of Agreement	-1.689	-2.068	-1.309	-48.49	-59.50	-37.51	-0.332	-0.414	-0.249	-2.285	-2.873	-1.696	-0.126	-0.158	-0.095
		Upper Limit of Agreement	1.500	1.121	1.880	43.82	32.80	54.80	0.361	0.279	0.443	2.662	2.074	3.251	0.139	0.108	0.171
	U	Mean Bias	-0.814	-1.302	-0.327	-15.80	-26.60	-5.11	0.009	-0.068	0.086	1.670	0.682	2.670	0.097	0.039	0.154
		Lower Limit of Agreement	-3.129	-3.680	-2.578	-66.90	-79.00	-54.72	-0.355	-0.441	-0.268	-3.040	-4.156	-1.920	-0.177	-0.243	-0.112
		Upper Limit of Agreement	1.501	0.950	2.051	35.20	23.00	47.29	0.372	0.286	0.459	6.380	5.264	7.510	0.371	0.306	0.436
	R	Mean Bias	0.247	-0.189	0.683	0.30	-7.70	8.31	0.030	-0.033	0.093	0.049	-0.355	0.452	-0.005	-0.029	0.018
		Lower Limit of Agreement	-1.822	-2.314	-1.330	-37.70	-46.74	-28.66	-0.269	-0.341	-0.198	-1.868	-2.324	-1.412	-0.115	-0.141	-0.089
		Upper Limit of Agreement	2.316	1.824	2.808	38.31	29.27	47.35	0.329	0.258	0.400	1.965	1.509	2.421	0.104	0.078	0.131
U2	L	Mean Bias	-0.476	-1.140	0.193	-8.25	-24.00	7.46	-0.017	-0.103	0.069	0.245	-0.425	0.915	0.010	-0.028	0.049
		Lower Limit of Agreement	-3.650	-4.410	-2.895	-82.83	-100.60	-65.09	-0.426	-0.523	-0.328	-2.936	-3.692	-2.179	-0.173	-0.216	-0.129
		Upper Limit of Agreement	2.699	1.940	3.454	66.33	48.60	84.07	0.391	0.294	0.488	3.425	2.669	4.182	0.193	0.150	0.237
	U	Mean Bias	-0.893	-1.650	-0.137	-11.10	-17.90	-4.28	-0.115	-0.226	-0.005	0.916	0.104	1.730	0.038	-0.015	0.091
		Lower Limit of Agreement	-4.487	-5.340	-3.632	-43.30	-51.00	-35.65	-0.639	-0.763	-0.514	-2.941	-3.859	-2.020	-0.214	-0.274	-0.154
		Upper Limit of Agreement	2.700	1.850	3.555	21.20	13.50	28.85	0.408	0.284	0.533	4.773	3.855	5.690	0.290	0.230	0.350
	R	Mean Bias	-0.172	-0.688	0.345	-5.56	-16.20	5.12	0.000	-0.086	0.086	0.204	-0.306	0.714	0.002	-0.028	0.031
		Lower Limit of Agreement	-2.624	-3.207	-2.041	-56.29	-68.40	-44.22	-0.408	-0.506	-0.311	-2.218	-2.794	-1.642	-0.139	-0.173	-0.106
		Upper Limit of Agreement	2.281	1.698	2.864	45.17	33.10	57.24	0.409	0.312	0.506	2.626	2.050	3.202	0.143	0.109	0.176
U3	L	Mean Bias	-0.241	-0.706	0.225	-6.54	-16.40	3.26	-0.015	-0.091	0.060	0.226	-0.257	0.708	0.005	-0.023	0.034
		Lower Limit of Agreement	-2.449	-2.975	-1.924	-53.11	-64.20	-42.03	-0.372	-0.457	-0.287	-2.066	-2.611	-1.521	-0.128	-0.160	-0.096
		Upper Limit of Agreement	1.968	1.443	2.494	40.02	28.90	51.10	0.342	0.257	0.427	2.517	1.972	3.062	0.139	0.107	0.171
	U	Mean Bias	-0.778	-1.520	-0.041	-13.30	-23.50	-3.18	-0.026	-0.137	0.086	1.150	0.325	1.970	0.059	0.010	0.108
		Lower Limit of Agreement	-4.278	-5.110	-3.446	-61.60	-73.00	-50.08	-0.553	-0.679	-0.428	-2.760	-3.689	-1.830	-0.174	-0.229	-0.119
		Upper Limit of Agreement	2.723	1.890	3.555	34.90	23.40	46.35	0.502	0.376	0.628	5.060	4.126	5.990	0.292	0.236	0.347
	R	Mean Bias	0.130	-0.187	0.446	1.63	-5.40	8.66	0.014	-0.089	0.117	-0.036	-0.465	0.393	-0.004	-0.030	0.022
		Lower Limit of Agreement	-1.373	-1.730	-1.015	-31.76	-39.70	-23.81	-0.475	-0.591	-0.358	-2.073	-2.557	-1.588	-0.129	-0.159	-0.099
		Upper Limit of Agreement	1.632	1.274	1.989	35.02	27.08	42.96	0.503	0.387	0.619	2.201	1.516	2.485	0.121	0.091	0.151

D1	L	Mean Bias	0.087	-0.416	0.589	-2.67	-11.00	5.63	0.003	-0.075	0.082	0.309	-0.218	0.835	0.011	-0.020	0.041
		Lower Limit of Agreement	-2.299	-2.867	-1.731	-42.08	-51.50	-32.70	-0.371	-0.460	-0.282	-2.191	-2.785	-1.596	-0.136	-0.170	-0.101
		Upper Limit of Agreement	2.472	1.905	3.040	36.73	27.40	46.11	0.378	0.289	0.467	2.808	2.214	3.403	0.157	0.122	0.192
	D	Mean Bias	-0.845	-1.430	-0.257	-19.30	-31.40	-7.14	-0.033	-0.149	0.084	1.320	0.491	2.140	0.068	0.017	0.119
		Lower Limit of Agreement	-3.636	-4.300	-2.972	-76.80	-90.50	-63.13	-0.587	-0.718	-0.455	-2.600	-3.530	-1.670	-0.176	-0.233	-0.118
		Upper Limit of Agreement	1.946	1.280	2.610	38.30	24.60	51.99	0.521	0.389	0.653	5.230	4.298	6.160	0.311	0.253	0.369
	R	Mean Bias	0.016	-0.483	0.515	-6.78	-17.20	3.67	0.027	-0.078	0.132	0.137	-0.523	0.797	-0.006	-0.045	0.034
		Lower Limit of Agreement	-2.354	-2.918	-1.790	-56.38	-68.20	-44.58	-0.470	-0.588	-0.352	-2.998	-3.744	-2.252	-0.192	-0.236	-0.147
		Upper Limit of Agreement	2.386	1.822	2.950	42.83	31.00	54.63	0.524	0.406	0.642	3.272	2.526	4.018	0.181	0.136	0.225
D2	L	Mean Bias	-0.521	-1.060	0.015	-6.45	-19.20	6.33	-0.078	-0.183	0.027	0.391	-0.325	1.110	0.020	-0.018	0.058
		Lower Limit of Agreement	-3.063	-3.670	-2.459	-67.12	-81.50	-52.68	-0.578	-0.697	-0.459	-3.011	-3.821	-2.200	-0.161	-0.204	-0.118
		Upper Limit of Agreement	2.022	1.420	2.627	54.22	39.80	68.65	0.422	0.303	0.541	3.794	2.984	4.600	0.201	0.158	0.244
	D	Mean Bias	-1.060	-2.450	0.316	-25.90	-46.30	-5.46	-0.146	-0.277	-0.014	1.500	0.427	2.580	0.064	0.001	0.127
		Lower Limit of Agreement	-7.620	-9.180	-6.061	-122.80	-145.90	-99.77	-0.769	-0.917	-0.621	-3.600	-4.815	-2.390	-0.236	-0.307	-0.164
		Upper Limit of Agreement	5.490	3.930	7.049	71.10	48.00	94.14	0.478	0.330	0.626	6.600	5.390	7.820	0.363	0.292	0.434
	R	Mean Bias	-0.097	-0.579	0.384	1.86	-7.12	10.80	0.039	-0.083	0.161	0.024	-0.511	0.559	-2.40e−4	-0.033	0.032
		Lower Limit of Agreement	-2.384	-2.927	-1.840	-40.79	-50.93	-30.60	-0.539	-0.676	-0.401	-2.518	-3.123	-1.914	-0.155	-0.192	-0.118
		Upper Limit of Agreement	2.189	1.645	2.733	44.51	34.37	54.70	0.617	0.480	0.750	2.566	1.962	3.171	0.154	0.118	0.191
D3	L	Mean Bias	-0.050	-0.558	0.458	-4.58	-15.30	6.17	-0.042	-0.142	0.058	0.035	-0.559	0.629	-0.008	-0.043	0.026
		Lower Limit of Agreement	-2.462	-3.036	-1.888	-55.64	-67.80	-43.49	-0.518	-0.631	-0.405	-2.785	-3.456	-2.114	-0.171	-0.210	-0.132
		Upper Limit of Agreement	2.361	1.788	2.935	46.47	34.30	58.00	0.434	0.320	0.547	2.855	2.184	3.526	0.154	0.115	0.193
	D	Mean Bias	-0.417	-1.270	0.436	-7.72	-19.20	3.79	-0.076	-0.188	0.037	0.597	-0.017	1.210	0.021	-0.023	0.065
		Lower Limit of Agreement	-4.467	-5.430	-3.503	-62.35	-75.40	-49.36	-0.611	-0.738	-0.483	-2.318	-3.012	-1.620	-0.186	-0.236	-0.137
		Upper Limit of Agreement	3.632	2.670	4.596	46.91	33.90	59.91	0.459	0.332	0.586	3.512	2.818	4.210	0.228	0.179	0.278
	R	Mean Bias	0.523	-0.061	1.110	11.30	-1.24	23.90	0.104	-7.33e−4	0.209	-0.534	-1.140	0.071	-0.021	-0.052	0.011
		Lower Limit of Agreement	-2.248	-2.907	-1.590	-48.40	-62.57	-34.20	-0.394	-0.512	-0.275	-3.407	-4.090	-2.723	-0.172	-0.208	-0.136
		Upper Limit of Agreement	3.294	2.635	3.950	71.00	56.82	85.20	0.602	0.483	0.720	2.338	1.650	3.021	0.131	0.095	0.167

U1-U3, D1-D3, measurement points on the scar; L, R, U, D, direction of measurement, left, right, up, down, respectively; F-MYO, myotonometric frequency, S-MYO, myotonometric stiffness, D-MYO, myotonometric decrement, R-MYO, myotonometric relaxation time, C-MYO, myotonometric creep; CI, confidence interval.
