# Supplementary material for: Inter‐ and Intra‐Rater Reliability of Myotonometric Assessment of the Mechanical Properties of Caesarean Section Scar Skin Using the MyotonPRO With an L‐Shaped Probe
Source: Skin Res Technol. 2026 Jan 9;32(1):e70315. doi: 10.1111/srt.70315 (PMC12784373; doi:10.1111/srt.70315)
Supplement: Supplementary file 4 — Table A.4. Numerical bias values and corresponding 95% limits of agreement for intra‐rater measurements obtained by rater R2. [file SRT-32-e70315-s001.pdf]

Table A.4. Numerical bias values and corresponding 95% limits of agreement for intra-rater measurements obtained by rater R2.

		Bland-Altman metrics	F-MYO			S-MYO			D-MYO			R-MYO			C-MYO		
			Estimate	Lower CI	Upper CI	Estimate	Lower CI	Upper CI	Estimate	Lower CI	Upper CI	Estimate	Lower CI	Upper CI	Estimate	Lower CI	Upper CI
U1	L	Mean Bias	-0.291	-0.805	0.224	-3.80	-15.40	7.78	0.073	-0.005	0.151	0.186	-0.374	0.747	0.014	-0.018	0.047
		Lower Limit of Agreement	-2.733	-3.315	-2.152	-58.80	-71.90	-45.72	-0.299	-0.387	-0.210	-2.476	-3.110	-1.843	-0.140	-0.176	-0.103
		Upper Limit of Agreement	2.152	1.571	2.733	51.20	38.10	64.29	0.445	0.356	0.533	2.849	2.216	3.483	0.168	0.131	0.205
	U	Mean Bias	-1.090	-1.760	-0.426	-18.60	-29.80	-7.46	0.089	-0.017	0.195	1.830	0.839	2.830	0.109	0.049	0.169
		Lower Limit of Agreement	-4.270	-5.020	-3.511	-71.80	-84.40	-59.15	-0.413	-0.532	-0.294	-2.890	-4.017	-1.770	-0.176	-0.243	-0.108
		Upper Limit of Agreement	2.080	1.320	2.833	34.50	21.90	47.14	0.591	0.471	0.710	6.560	5.437	7.690	0.395	0.327	0.462
	R	Mean Bias	-0.432	-1.270	0.405	-11.30	-31.20	8.65	0.007	-0.062	0.076	0.250	-0.355	0.855	0.013	-0.022	0.048
		Lower Limit of Agreement	-4.403	-5.350	-3.458	-106.00	-128.50	-83.46	-0.322	-0.400	-0.244	-2.623	-3.307	-1.940	-0.151	-0.190	-0.112
		Upper Limit of Agreement	3.540	2.600	4.484	83.40	60.90	105.92	0.336	0.258	0.414	3.122	2.439	3.806	0.177	0.138	0.216
U2	L	Mean Bias	-0.250	-1.210	0.712	-10.90	-30.30	8.50	0.027	-0.058	0.112	0.156	-0.417	0.729	0.004	-0.028	0.036
		Lower Limit of Agreement	-4.816	-5.900	-3.730	-103.10	-125.00	-81.18	-0.376	-0.472	-0.280	-2.565	-3.212	-1.918	-0.149	-0.185	-0.113
		Upper Limit of Agreement	4.316	3.230	5.402	81.30	59.30	103.20	0.430	0.334	0.526	2.877	2.230	3.524	0.157	0.120	0.193
	U	Mean Bias	0.451	-0.621	1.520	-5.68	-12.70	1.37	0.056	-0.030	0.141	0.409	-0.292	1.110	0.022	-0.022	0.065
		Lower Limit of Agreement	-4.639	-5.850	-3.430	-39.14	-47.10	-31.18	-0.350	-0.447	-0.254	-2.918	-3.710	-2.130	-0.186	-0.235	-0.136
		Upper Limit of Agreement	5.541	4.330	6.750	27.78	19.80	35.74	0.461	0.365	0.558	3.736	2.944	4.530	0.229	0.180	0.278
	R	Mean Bias	-0.396	-1.310	0.522	-11.80	-31.80	8.19	-0.009	-0.095	0.077	0.248	-0.375	0.871	0.010	-0.025	0.044
		Lower Limit of Agreement	-4.755	-5.790	-3.718	-106.90	-129.50	-84.26	-0.416	-0.513	-0.319	-2.708	-3.411	-2.005	-0.156	-0.196	-0.117
		Upper Limit of Agreement	3.963	2.930	4.999	83.20	60.60	105.83	0.398	0.301	0.495	3.204	2.501	3.907	0.175	0.136	0.215
U3	L	Mean Bias	-0.282	-0.934	0.369	-5.38	-16.20	5.44	-0.021	-0.117	0.075	0.034	-0.426	0.495	0.004	-0.026	0.035
		Lower Limit of Agreement	-3.377	-4.113	-2.641	-56.80	-69.00	-44.57	-0.478	-0.587	-0.369	-2.151	-2.671	-1.631	-0.138	-0.172	-0.104
		Upper Limit of Agreement	2.812	2.076	3.548	46.03	33.80	58.26	0.436	0.327	0.545	2.220	1.700	2.740	0.147	0.113	0.181
	U	Mean Bias	-0.973	-1.850	-0.095	-11.20	-23.80	1.35	0.053	-0.059	0.165	1.530	0.368	2.680	0.093	0.023	0.163
		Lower Limit of Agreement	-5.139	-6.130	-4.148	-70.90	-85.10	-56.72	-0.479	-0.605	-0.352	-3.970	-5.278	-2.660	-0.238	-0.317	-0.159
		Upper Limit of Agreement	3.194	2.200	4.185	48.50	34.30	62.68	0.584	0.458	0.711	7.020	5.714	8.330	0.425	0.346	0.503
	R	Mean Bias	0.167	-0.261	0.596	-0.26	-9.49	8.98	-0.031	-0.135	0.073	-0.055	-0.477	0.367	-0.001	-0.024	0.021
		Lower Limit of Agreement	-1.867	-2.351	-1.383	-44.09	-54.52	-33.66	-0.523	-0.640	-0.406	-2.059	-2.535	-1.582	-0.106	-0.131	-0.081
		Upper Limit of Agreement	2.201	1.717	2.685	43.58	33.15	54.00	0.462	0.344	0.579	1.948	1.472	2.425	0.103	0.078	0.128

D1	L	Mean Bias	-0.143	-0.691	0.405	-2.91	-16.50	10.70	0.030	-0.072	0.133	0.554	-0.181	1.290	0.035	-0.008	0.077
		Lower Limit of Agreement	-2.746	-3.366	-2.127	-67.44	-82.80	-52.10	-0.456	-0.572	-0.341	-2.935	-3.765	-2.110	-0.168	-0.216	-0.120
		Upper Limit of Agreement	2.460	1.841	3.079	61.62	46.30	77.00	0.517	0.401	0.632	4.044	3.214	4.870	0.237	0.189	0.286
	D	Mean Bias	-1.310	-2.530	-0.080	-26.70	-49.90	-3.57	0.001	-0.129	0.130	1.440	0.176	2.700	0.068	-0.007	0.144
		Lower Limit of Agreement	-7.140	-8.520	-5.749	-136.60	-162.80	-110.49	-0.615	-0.761	-0.468	-4.550	-5.980	-3.130	-0.292	-0.378	-0.206
		Upper Limit of Agreement	4.520	3.130	5.907	83.20	57.00	109.34	0.616	0.469	0.762	7.430	6.006	8.860	0.429	0.343	0.514
	R	Mean Bias	0.117	-0.467	0.701	-0.17	-14.90	14.60	-0.013	-0.104	0.079	0.154	-0.525	0.834	0.005	-0.036	0.045
		Lower Limit of Agreement	-2.657	-3.317	-1.997	-70.05	-86.70	-53.40	-0.449	-0.552	-0.345	-3.071	-3.838	-2.303	-0.188	-0.234	-0.142
		Upper Limit of Agreement	2.891	2.231	3.551	69.72	53.10	86.30	0.424	0.320	0.527	3.379	2.612	4.147	0.197	0.152	0.243
D2	L	Mean Bias	0.162	-0.869	1.190	1.41	-20.10	22.90	-0.101	-0.231	0.030	0.334	-0.368	1.030	0.015	-0.026	0.056
		Lower Limit of Agreement	-4.733	-5.897	-3.570	-100.65	-124.90	-76.40	-0.720	-0.868	-0.573	-2.996	-3.788	-2.200	-0.182	-0.228	-0.135
		Upper Limit of Agreement	5.056	3.892	6.220	103.47	79.20	127.70	0.519	0.372	0.667	3.663	2.871	4.450	0.212	0.165	0.258
	D	Mean Bias	-1.070	-2.030	-0.105	-19.40	-32.30	-6.54	-0.091	-0.209	0.027	0.834	-0.301	1.970	0.026	-0.052	0.103
		Lower Limit of Agreement	-5.640	-6.720	-4.549	-80.60	-95.20	-66.07	-0.650	-0.783	-0.517	-4.554	-5.835	-3.270	-0.342	-0.430	-0.255
		Upper Limit of Agreement	3.500	2.420	4.589	41.80	27.20	56.33	0.468	0.335	0.601	6.221	4.939	7.500	0.393	0.306	0.481
	R	Mean Bias	0.366	-0.591	1.320	4.56	-18.20	27.30	-0.090	-0.197	0.018	0.194	-0.481	0.870	0.008	-0.032	0.048
		Lower Limit of Agreement	-4.179	-5.260	-3.100	-103.30	-129.00	-77.60	-0.600	-0.721	-0.479	-3.012	-3.775	-2.250	-0.183	-0.228	-0.137
		Upper Limit of Agreement	4.911	3.830	5.990	112.42	86.80	138.10	0.420	0.299	0.542	3.401	2.638	4.164	0.198	0.153	0.243
D3	L	Mean Bias	-0.049	-0.647	0.549	0.02	-14.40	14.50	-0.045	-0.142	0.051	-0.230	-0.938	0.478	-0.022	-0.062	0.018
		Lower Limit of Agreement	-2.887	-3.562	-2.212	-68.64	-85.00	-52.30	-0.502	-0.611	-0.393	-3.591	-4.391	-2.792	-0.213	-0.258	-0.167
		Upper Limit of Agreement	2.790	2.114	3.465	68.68	52.30	85.00	0.411	0.302	0.520	3.132	2.332	3.932	0.169	0.123	0.214
	D	Mean Bias	-0.477	-1.110	0.158	-12.80	-24.00	-1.71	-0.066	-0.162	0.031	0.740	0.062	1.420	0.034	-0.008	0.076
		Lower Limit of Agreement	-3.492	-4.210	-2.775	-65.70	-78.30	-53.14	-0.525	-0.635	-0.416	-2.478	-3.244	-1.710	-0.167	-0.214	-0.119
		Upper Limit of Agreement	2.537	1.820	3.254	40.00	27.40	52.60	0.394	0.285	0.504	3.958	3.193	4.720	0.234	0.187	0.282
	R	Mean Bias	0.417	-0.069	0.903	9.24	-0.62	19.10	0.054	-0.046	0.154	-0.567	-1.230	0.091	-0.028	-0.066	0.009
		Lower Limit of Agreement	-1.891	-2.440	-1.342	-37.57	-48.70	-26.40	-0.420	-0.533	-0.308	-3.691	-4.430	-2.948	-0.205	-0.247	-0.163
		Upper Limit of Agreement	2.725	2.176	3.274	56.05	44.91	67.20	0.528	0.415	0.641	2.556	1.810	3.299	0.149	0.107	0.191

U1-U3, D1-D3, measurement points on the scar; L, R, U, D, direction of measurement, left, right, up, down, respectively; F-MYO, myotonometric frequency, S-MYO, myotonometric stiffness, D-MYO, myotonometric decrement, R-MYO, myotonometric relaxation time, C-MYO, myotonometric creep; CI, confidence interval.
