# Supplementary material for: Inter‐ and Intra‐Rater Reliability of Myotonometric Assessment of the Mechanical Properties of Caesarean Section Scar Skin Using the MyotonPRO With an L‐Shaped Probe
Source: Skin Res Technol. 2026 Jan 9;32(1):e70315. doi: 10.1111/srt.70315 (PMC12784373; doi:10.1111/srt.70315)
Supplement: Supplementary file 5 — Table A.5. Inter‐rater comparison of MyotonPRO parameter values recorded during measurement session S1 [file SRT-32-e70315-s006.pdf]

Table A.5. Inter-rater comparison of MyotonPRO® parameter values recorded during measurement session S1.

Comparison parameters			Measurement points on the scar																	
			U1			U2			U3			D1			D2			D3		
			L	U	R	L	U	R	L	U	R	L	D	R	L	D	R	L	D	R
F-MYO [Hz]	R1	$\bar{x}$	15.82	15.97	16.67	17.06	14.26	17.57	16.66	15.55	16.28	16.42	15.91	16.64	17.00	14.44	17.26	17.08	15.69	16.85
		SD	1.35	3.09	2.72	1.65	2.79	2.99	1.54	2.95	1.28	2.50	2.49	3.05	3.29	1.99	3.69	2.72	2.67	2.67
	R2	$\bar{x}$	15.84	15.72	15.97	17.88	15.09	17.77	17.19	15.85	16.57	16.45	16.03	16.41	17.79	14.67	17.86	17.70	16.08	17.35
		SD	1.25	2.73	1.33	2.64	2.97	2.56	1.82	2.93	1.58	2.62	2.62	3.30	4.72	2.56	4.48	3.30	2.78	3.08
	p		0.90	0.22	0.07	0.01	0.13	0.28	0.01	0.16	0.12	0.82	0.39	0.07	0.03	0.50	0.01	0.00	0.22	0.06
	t		-0.13	1.25	1.91	-2.78	-1.56	-1.11	-2.99	-1.46	-1.62	-0.23	-0.87	1.90	-2.30	-0.68	-2.71	-3.19	-1.27	-1.96
S-MYO [N/m]	R1	$\bar{x}$	223.69	245.21	250.49	253.06	212.02	269.31	252.16	248.94	240.38	234.59	230.53	252.62	253.58	207.64	262.22	263.81	236.64	250.31
		SD	25.68	48.25	59.43	32.06	29.78	65.92	44.12	51.48	36.56	56.78	44.99	72.65	69.39	33.50	72.71	61.71	50.90	49.76
	R2	$\bar{x}$	225.41	245.18	239.54	265.58	212.79	272.83	268.19	254.33	246.47	236.91	231.41	251.35	269.62	214.94	278.70	279.07	245.76	258.02
		SD	25.59	46.62	31.68	54.51	29.39	57.69	52.41	51.49	38.58	68.44	48.31	83.86	105.89	40.01	103.68	79.69	54.81	65.75
	p		0.59	1.00	0.11	0.05	0.80	0.32	0.00	0.16	0.15	0.51	0.78	0.76	0.07	0.12	0.04	0.02	0.05	0.29
	t		-0.54	0.01	1.66	-2.07	-0.26	-1.02	-3.73	-1.44	-1.50	-0.67	-0.29	0.32	-1.87	-1.61	-2.13	-2.57	-2.11	-1.08
D-MYO [log]	R1	$\bar{x}$	1.81	1.95	1.85	2.02	1.91	2.03	1.84	1.98	1.89	1.64	1.88	1.57	1.78	1.61	1.86	1.59	1.80	1.83
		SD	0.24	0.34	0.21	0.23	0.29	0.32	0.26	0.39	0.23	0.23	0.42	0.26	0.25	0.42	0.36	0.28	0.40	0.34
	R2	$\bar{x}$	1.86	2.02	1.82	2.08	2.02	2.04	1.88	1.98	1.87	1.66	1.90	1.53	1.79	1.69	1.81	1.58	1.85	1.78
		SD	0.24	0.35	0.22	0.26	0.41	0.26	0.30	0.37	0.23	0.27	0.42	0.25	0.27	0.42	0.28	0.25	0.39	0.30
	p		0.10	0.07	0.40	0.03	0.01	0.54	0.12	0.92	0.45	0.48	0.20	0.29	0.76	0.04	0.23	0.88	0.16	0.24
	t		-1.71	-1.88	0.86	-2.25	-2.96	-0.62	-1.62	-0.10	0.77	-0.72	-1.31	1.09	-0.31	-2.20	1.23	0.16	-1.45	1.20
R-MYO [ms]	R1	$\bar{x}$	21.17	20.93	19.75	19.22	23.30	18.64	19.27	21.27	20.13	20.44	21.55	19.27	19.27	23.04	18.79	18.45	21.10	19.44
		SD	1.88	4.38	2.66	1.84	2.73	2.68	2.34	4.82	1.94	2.70	3.07	2.82	2.84	2.63	2.72	2.81	3.38	2.63
	R2	$\bar{x}$	20.89	21.27	20.21	18.52	23.13	18.38	18.45	20.81	19.84	20.34	21.19	19.59	18.65	22.50	18.21	17.60	20.51	18.85
		SD	1.84	4.19	1.98	2.36	2.82	2.51	2.60	4.37	2.22	2.86	2.96	3.11	3.24	2.93	3.04	2.93	3.29	2.79
	p		0.20	0.29	0.08	0.01	0.57	0.14	< .001	0.30	0.20	0.55	0.21	0.12	0.02	0.06	0.01	< .001	0.03	0.08
	t		1.33	-1.09	-1.86	3.06	0.58	1.52	4.05	1.06	1.32	0.61	1.28	-1.64	2.45	2.02	3.08	3.88	2.39	1.87
C-MYO [De]	R1	$\bar{x}$	1.25	1.25	1.18	1.15	1.38	1.12	1.14	1.28	1.19	1.20	1.28	1.13	1.14	1.35	1.11	1.09	1.25	1.15
		SD	0.10	0.25	0.14	0.11	0.17	0.15	0.12	0.28	0.09	0.15	0.16	0.15	0.15	0.16	0.15	0.14	0.19	0.13
	R2	$\bar{x}$	1.23	1.28	1.21	1.11	1.37	1.11	1.11	1.26	1.19	1.20	1.25	1.16	1.11	1.32	1.09	1.04	1.23	1.12
		SD	0.10	0.24	0.11	0.13	0.18	0.14	0.14	0.26	0.12	0.16	0.16	0.17	0.18	0.18	0.17	0.15	0.18	0.15
	p		0.29	0.13	0.03	0.01	0.64	0.53	0.01	0.39	0.74	0.94	0.19	0.13	0.08	0.10	0.07	0.00	0.09	0.15
	t		1.09	-1.56	-2.34	2.70	0.47	0.64	2.99	0.87	0.34	0.08	1.35	-1.55	1.86	1.71	1.89	3.35	1.76	1.48

U1-U3, D1-D3, measurement points on the scar; L, R, U, D, direction of measurement, left, right, up, down, respectively; F-MYO, myotonometric frequency; S-MYO, myotonometric stiffness; D-MYO, myotonometric decrement; R-MYO, myotonometric relaxation time; C-MYO, myotonometric creep; R1, rater 1; R2, rater 2; $\bar{x}$, mean; SD, standard deviation; p, p-value; t, the ratio of the difference.
