# Supplementary material for: Inter‐ and Intra‐Rater Reliability of Myotonometric Assessment of the Mechanical Properties of Caesarean Section Scar Skin Using the MyotonPRO With an L‐Shaped Probe
Source: Skin Res Technol. 2026 Jan 9;32(1):e70315. doi: 10.1111/srt.70315 (PMC12784373; doi:10.1111/srt.70315)
Supplement: Supplementary file 6 — Table A.6. Inter‐rater comparison of MyotonPRO parameter values recorded during measurement session S2. [file SRT-32-e70315-s005.pdf]

Table A.6. Inter-rater comparison of MyotonPRO® parameter values recorded during measurement session S2.

Comparison parameters			Measurement points on the scar																	
			U1			U2			U3			D1			D2			D3		
			L	U	R	L	U	R	L	U	R	L	D	R	L	D	R	L	D	R
F-MYO [Hz]	R1	$\bar{x}$	15.91	16.78	16.42	17.54	15.16	17.74	16.90	16.33	16.15	16.33	16.75	16.63	17.52	15.51	17.36	17.13	16.11	16,33
		SD	1.49	2.92	2.02	2.72	2.85	3.45	1.99	2.87	1.36	2.07	2.51	2.95	3.34	3.36	3.62	2.66	2.31	2,62
	R2	$\bar{x}$	16.13	16.82	16.40	18.13	14.64	18.16	17.47	16.82	16.40	16.60	17.34	16.30	17.63	15.74	17.49	17.75	16.56	16,93
		SD	1.93	2.82	2.83	4.24	2.39	4.09	2.68	2.86	2.17	2.42	3.39	2.34	2.81	3.23	2.75	2.93	2.77	3,27
	p		0.36	0.82	0.95	0.11	0.11	0.03	0.03	0.13	0.25	0.25	0.18	0.06	0.58	0.65	0.67	< .001	0.06	0.01
	t		3.12	0.43	-1.94	3.82	0.56	1.17	1.17	2.39	-0.07	2.32	1.69	0.94	2.00	0.45	1.38	1.57	-1.68	0.23
S-MYO [N/m]	R1	$\bar{x}$	226.02	261.06	250.18	261.31	223.09	274.87	258.70	262.27	238.75	237.26	249.79	259.40	260.03	233.52	260.36	268.39	244.36	238,98
		SD	32.40	54.03	47.06	56.93	31.24	75.28	52.63	50.58	35.55	47.78	55.46	70.51	74.16	63.48	71.32	61.12	42.82	55,03
	R2	$\bar{x}$	229.21	263.83	250.83	276.50	218.47	284.66	273.58	265.55	246.73	239.82	258.13	251.52	268.22	234.38	274.14	279.06	258.61	248,78
		SD	46.42	50.06	73.24	91.43	31.23	95.19	67.93	48.74	51.17	53.66	69.07	59.47	61.78	53.97	60.26	68.44	49.99	66,62
	p		0.49	0.45	0.92	0.07	0.05	0.04	0.01	0.50	0.14	0.43	0.33	0.04	0.04	0.88	0.01	0.01	0.00	0.01
	t		0.70	0.78	0.10	1.94	-2.09	2.13	2.73	0.69	1.52	0.80	0.99	-2.21	2.20	0.16	2.72	2.69	3.67	2.78
D-MYO [log]	R1	$\bar{x}$	1.79	1.94	1.82	2.03	2.03	2.03	1.85	2.01	1.88	1.64	1.91	1.54	1.86	1.76	1.82	1.63	1.87	1,72
		SD	0.26	0.29	0.18	0.27	0.36	0.29	0.23	0.35	0.27	0.18	0.37	0.20	0.32	0.35	0.26	0.23	0.38	0,29
	R2	$\bar{x}$	1.78	1.93	1.81	2.05	1.96	2.05	1.90	1.93	1.90	1.63	1.90	1.54	1.89	1.78	1.90	1.63	1.91	1,73
		SD	0.24	0.31	0.18	0.22	0.31	0.32	0.27	0.35	0.30	0.21	0.38	0.23	0.30	0.35	0.29	0.25	0.33	0,26
	p		0.78	0.67	0.97	0.54	0.10	0.41	0.10	0.06	0.59	0.97	0.80	0.94	0.28	0.43	0.08	0.98	0.16	0.92
	t		-0.28	-0.43	-0.04	0.63	-1.72	0.85	1.70	-1.98	0.55	-0.04	-0.26	0.07	1.10	0.80	1.85	-0.03	1.44	0.10
R-MYO [ms]	R1	$\bar{x}$	20.98	19.25	19.70	18.98	22.38	18.44	19.04	20.12	20.16	20.13	20.24	19.13	18.87	21.54	18.77	18.42	20.50	19,97
		SD	2.01	3.69	2.36	2.47	2.78	2.68	2.57	4.55	1.92	2.59	3.52	2.91	2.82	3.61	2.60	2.65	3.25	2,62
	R2	$\bar{x}$	20.70	19.44	19.96	18.36	22.73	18.13	18.41	19.28	19.90	19.79	19.75	19.44	18.31	21.67	18.01	17.83	19.77	19,42
		SD	2.59	3.67	2.81	2.94	2.75	2.96	2.81	4.11	2.52	2.74	3.81	2.86	2.60	3.51	2.41	2.99	3.50	2,95
	p		0.31	0.42	0.24	0.00	0.09	0.03	0.00	0.06	0.20	0.10	0.15	0.08	0.01	0.62	0.00	0.00	0.01	0.00
	t		-1.04	0.81	1.21	-3.55	1.78	-2.36	-3.26	-1.95	-1.31	-1.73	-1.47	1.83	-2.91	0.51	-3.61	-3.20	-2.97	-3.40
C-MYO [De]	R1	$\bar{x}$	1.24	1.16	1.18	1.14	1.34	1.12	1.14	1.22	1.2	1.19	1.21	1.14	1.12	1.28	1.12	1.1	1.23	1,17
		SD	0.1	0.21	0.13	0.14	0.17	0.15	0.13	0.27	0.1	0.14	0.19	0.16	0.15	0.21	0.14	0.14	0.17	0,13
	R2	$\bar{x}$	1.22	1.17	1.19	1.11	1.35	1.1	1.11	1.17	1.19	1.17	1.18	1.15	1.1	1.3	1.08	1.07	1.19	1,15
		SD	0.14	0.21	0.16	0.17	0.17	0.17	0.15	0.24	0.13	0.14	0.2	0.15	0.14	0.21	0.13	0.16	0.19	0,15
	p		0.18	0.22	0.32	0.00	0.45	0.05	0.01	0.04	0.60	0.04	0.16	0.21	0.08	0.52	0.02	0.01	0.02	0.07
	t		-1.39	1.25	1.01	-3.25	0.77	-2.07	-3.03	-2.20	-0.53	-2.17	-1.46	1.28	-1.86	0.65	-2.52	-2.98	-2.53	-1.89

U1-U3, D1-D3, measurement points on the scar; L, R, U, D, direction of measurement, left, right, up, down, respectively; F-MYO, myotonometric frequency, S-MYO, myotonometric stiffness, D-MYO, myotonometric decrement, R-MYO, myotonometric relaxation time, C-MYO, myotonometric creep; R1, rater 1; R2, rater 2; $\bar{x}$, mean; SD, standard deviation; p, p-value; t, the ratio of the difference.
