# Supplementary material for: Inter‐ and Intra‐Rater Reliability of Myotonometric Assessment of the Mechanical Properties of Caesarean Section Scar Skin Using the MyotonPRO With an L‐Shaped Probe
Source: Skin Res Technol. 2026 Jan 9;32(1):e70315. doi: 10.1111/srt.70315 (PMC12784373; doi:10.1111/srt.70315)
Supplement: Supplementary file 7 — Table A.7. Comparison of MyotonPRO parameter values across sessions performed by rater R1 [file SRT-32-e70315-s002.pdf]

Table A.7. Comparison of MyotonPRO® parameter values across sessions performed by rater R1.

Comparison parameters			Measurement points on the scar																	
			U1			U2			U3			D1			D2			D3		
			L	U	R	L	U	R	L	U	R	L	D	R	L	D	R	L	D	R
F-MYO [Hz]	S1	$\bar{x}$	15.82	15.97	16.67	17.06	14.26	17.57	16.66	15.55	16.28	16.42	15.91	16.64	17.00	14.44	17.26	17.08	15.69	16,85
		SD	1.35	3.09	2.72	1.65	2.79	2.99	1.54	2.95	1.28	2.50	2.49	3.05	3.29	1.99	3.69	2.72	2.67	2,67
	S2	$\bar{x}$	15.91	16.78	16.42	17.54	15.16	17.74	16.90	16.33	16.15	16.33	16.75	16.63	17.52	15.51	17.36	17.13	16.11	16,33
		SD	1.49	2.92	2.02	2.72	2.85	3.45	1.99	2.87	1.36	2.07	2.51	2.95	3.34	3.36	3.62	2.66	2.31	2,62
	p		0.57	0.00	0.25	0.16	0.02	0.50	0.30	0.04	0.41	0.73	0.01	0.95	0.06	0.12	0.68	0.84	0.32	0.08
	t		-0.58	-3.45	1.17	-1.47	-2.44	-0.69	-1.07	-2.18	0.85	0.36	-2.97	0.07	-2.01	-1.59	-0.42	-0.20	-1.01	1.85
S-MYO [N/m]	S1	$\bar{x}$	223.69	245.21	250.49	253.06	212.02	269.31	252.16	248.94	240.38	234.59	230.53	252.62	253.58	207.64	262.22	263.81	236.64	250,31
		SD	25.68	48.25	59.43	32.06	29.78	65.92	44.12	51.48	36.56	56.78	44.99	72.65	69.39	33.50	72.71	61.71	50.90	49,76
	S2	$\bar{x}$	226.02	261.06	250.18	261.31	223.09	274.87	258.70	262.27	238.75	237.26	249.79	259.40	260.03	233.52	260.36	268.39	244.36	238,98
		SD	32.40	54.03	47.06	56.93	31.24	75.28	52.63	50.58	35.55	47.78	55.46	70.51	74.16	63.48	71.32	61.12	42.82	55,03
	p		0.62	0.01	0.94	0.29	0.00	0.29	0.18	0.01	0.64	0.51	0.00	0.19	0.31	0.02	0.67	0.39	0.18	0.08
	t		-0.50	-3.05	0.08	-1.08	-3.37	-1.07	-1.38	-2.71	0.48	-0.66	-3.28	-1.34	-1.04	-2.62	0.43	-0.88	-1.39	1.86
D-MYO [log]	S1	$\bar{x}$	1.81	1.95	1.85	2.02	1.91	2.03	1.84	1.98	1.89	1.64	1.88	1.57	1.78	1.61	1.86	1.59	1.80	1,83
		SD	0.24	0.34	0.21	0.23	0.29	0.32	0.26	0.39	0.23	0.23	0.42	0.26	0.25	0.42	0.36	0.28	0.40	0,34
	S2	$\bar{x}$	1.79	1.94	1.82	2.03	2.03	2.03	1.85	2.01	1.88	1.64	1.91	1.54	1.86	1.76	1.82	1.63	1.87	1,72
		SD	0.26	0.29	0.18	0.27	0.36	0.29	0.23	0.35	0.27	0.18	0.37	0.20	0.32	0.35	0.26	0.23	0.38	0,29
	p		0.68	0.81	0.34	0.68	0.04	1.00	0.68	0.64	0.78	0.93	0.57	0.60	0.14	0.03	0.51	0.40	0.18	0.05
	t		0.41	0.24	0.98	-0.42	-2.16	0.00	-0.42	-0.48	0.28	0.09	-0.58	0.53	-1.53	-2.29	0.67	-0.87	-1.39	2.05
R-MYO [ms]	S1	$\bar{x}$	21.17	20.93	19.75	19.22	23.30	18.64	19.27	21.27	20.13	20.44	21.55	19.27	19.27	23.04	18.79	18.45	21.10	19,44
		SD	1.88	4.38	2.66	1.84	2.73	2.68	2.34	4.82	1.94	2.70	3.07	2.82	2.84	2.63	2.72	2.81	3.38	2,63
	S2	$\bar{x}$	20.98	19.25	19.70	18.98	22.38	18.44	19.04	20.12	20.16	20.13	20.24	19.13	18.87	21.54	18.77	18.42	20.50	19,97
		SD	2.01	3.69	2.36	2.47	2.78	2.68	2.57	4.55	1.92	2.59	3.52	2.91	2.82	3.61	2.60	2.65	3.25	2,62
	p		0.46	0.00	0.81	0.46	0.03	0.42	0.34	0.01	0.86	0.24	0.00	0.67	0.27	0.01	0.93	0.90	0.06	0.08
	t		0.75	3.48	0.25	0.75	2.33	0.83	0.97	2.88	-0.17	1.21	3.29	0.43	1.13	2.88	0.09	0.12	2.01	-1.82
C-MYO [De]	S1	$\bar{x}$	1.25	1.25	1.18	1.15	1.38	1.12	1.14	1.28	1.19	1.20	1.28	1.13	1.14	1.35	1.11	1.09	1.25	1,15
		SD	0.10	0.25	0.14	0.11	0.17	0.15	0.12	0.28	0.09	0.15	0.16	0.15	0.15	0.16	0.15	0.14	0.19	0,13
	S2	$\bar{x}$	1.24	1.16	1.18	1.14	1.34	1.12	1.14	1.22	1.20	1.19	1.21	1.14	1.12	1.28	1.12	1.10	1.23	1,17
		SD	0.10	0.21	0.13	0.14	0.17	0.15	0.13	0.27	0.10	0.14	0.19	0.16	0.15	0.21	0.14	0.14	0.17	0,13
	p		0.63	0.00	0.64	0.59	0.15	0.91	0.69	0.02	0.76	0.48	0.01	0.77	0.29	0.05	0.99	0.62	0.33	0.20
	t		0.49	3.46	-0.48	0.55	1.48	0.12	0.40	2.47	-0.31	0.71	2.73	-0.29	1.09	2.08	-0.02	-0.51	0.99	-1.33

U1-U3, D1-D3, measurement points on the scar; L, R, U, D, direction of measurement, left, right, up, down, respectively; F-MYO, myotonometric frequency, S-MYO, myotonometric stiffness, D-MYO, myotonometric decrement, R-MYO, myotonometric relaxation time, C-MYO, myotonometric creep; S1, measuring session 1; S2, measuring session 2; $\bar{x}$, mean; SD, standard deviation; p, p-value; t, the ratio of the difference.
