# Supplementary material for: Inter‐ and Intra‐Rater Reliability of Myotonometric Assessment of the Mechanical Properties of Caesarean Section Scar Skin Using the MyotonPRO With an L‐Shaped Probe
Source: Skin Res Technol. 2026 Jan 9;32(1):e70315. doi: 10.1111/srt.70315 (PMC12784373; doi:10.1111/srt.70315)
Supplement: Supplementary file 8 — Table A.8. Comparison of MyotonPRO parameter values across sessions performed by rater R2. [file SRT-32-e70315-s004.pdf]

Table A.8. Comparison of MyotonPRO® parameter values across sessions performed by rater R2.

| Comparison parameters | | | Measurement points on the scar | | | | | | | | | | | | | | | | | |
|---|---|---|---|---|---|---|---|---|---|---|---|---|---|---|---|---|---|---|---|---|
| | | | U1 | | | U2 | | | U3 | | | D1 | | | D2 | | | D3 | | |
| | | | L | U | R | L | U | R | L | U | R | L | D | R | L | D | R | L | D | R |
| F-MYO [Hz] | S1 | $\bar{x}$ | 15.84 | 15.72 | 15.97 | 17.88 | 15.09 | 17.77 | 17.19 | 15.85 | 16.57 | 16.45 | 16.03 | 16.41 | 17.79 | 14.67 | 17.86 | 17.70 | 16.08 | 17,35 |
| | | SD | 1.25 | 2.73 | 1.33 | 2.64 | 2.97 | 2.56 | 1.82 | 2.93 | 1.58 | 2.62 | 2.62 | 3.30 | 4.72 | 2.56 | 4.48 | 3.30 | 2.78 | 3,08 |
| | S2 | $\bar{x}$ | 16.13 | 16.82 | 16.40 | 18.13 | 14.64 | 18.16 | 17.47 | 16.82 | 16.40 | 16.60 | 17.34 | 16.30 | 17.63 | 15.74 | 17.49 | 17.75 | 16.56 | 16,93 |
| | | SD | 1.93 | 2.82 | 2.83 | 4.24 | 2.39 | 4.09 | 2.68 | 2.86 | 2.17 | 2.42 | 3.39 | 2.34 | 2.81 | 3.23 | 2.75 | 2.93 | 2.77 | 3,27 |
| | p | | 0.26 | 0.00 | 0.30 | 0.60 | 0.39 | 0.38 | 0.38 | 0.03 | 0.43 | 0.59 | 0.04 | 0.68 | 0.75 | 0.03 | 0.44 | 0.87 | 0.13 | 0.09 |
| | t | | -1.16 | -3.37 | -1.06 | -0.54 | 0.87 | -0.89 | -0.90 | -2.28 | 0.81 | -0.54 | -2.20 | 0.42 | 0.32 | -2.29 | 0.79 | -0.17 | -1.55 | 1.76 |
| S-MYO [N/m] | S1 | $\bar{x}$ | 225.41 | 245.18 | 239.54 | 265.58 | 212.79 | 272.83 | 268.19 | 254.33 | 246.47 | 236.91 | 231.41 | 251.35 | 269.62 | 214.94 | 278.70 | 279.07 | 245.76 | 258,02 |
| | | SD | 25.59 | 46.62 | 31.68 | 54.51 | 29.39 | 57.69 | 52.41 | 51.49 | 38.58 | 68.44 | 48.31 | 83.86 | 105.89 | 40.01 | 103.68 | 79.69 | 54.81 | 65,75 |
| | S2 | $\bar{x}$ | 229.21 | 263.83 | 250.83 | 276.50 | 218.47 | 284.66 | 273.58 | 265.55 | 246.73 | 239.82 | 258.13 | 251.52 | 268.22 | 234.38 | 274.14 | 279.06 | 258.61 | 248,78 |
| | | SD | 46.42 | 50.06 | 73.24 | 91.43 | 31.23 | 95.19 | 67.93 | 48.74 | 51.17 | 53.66 | 69.07 | 59.47 | 61.78 | 53.97 | 60.26 | 68.44 | 49.99 | 66,62 |
| | p | | 0.51 | 0.00 | 0.25 | 0.26 | 0.11 | 0.24 | 0.32 | 0.08 | 0.96 | 0.66 | 0.03 | 0.98 | 0.89 | 0.01 | 0.68 | 1.00 | 0.03 | 0.07 |
| | t | | -0.68 | -3.44 | -1.17 | -1.16 | -1.66 | -1.22 | -1.03 | -1.84 | -0.06 | -0.44 | -2.38 | -0.02 | 0.14 | -3.11 | 0.41 | 0.00 | -2.38 | 1.94 |
| D-MYO [log] | S1 | $\bar{x}$ | 1.86 | 2.02 | 1.82 | 2.08 | 2.02 | 2.04 | 1.88 | 1.98 | 1.87 | 1.66 | 1.90 | 1.53 | 1.79 | 1.69 | 1.81 | 1.58 | 1.85 | 1,78 |
| | | SD | 0.24 | 0.41 | 0.22 | 0.26 | 0.35 | 0.26 | 0.30 | 0.37 | 0.23 | 0.27 | 0.42 | 0.25 | 0.27 | 0.42 | 0.28 | 0.25 | 0.39 | 0,30 |
| | S2 | $\bar{x}$ | 1.78 | 1.96 | 1.81 | 2.05 | 1.93 | 2.05 | 1.90 | 1.93 | 1.90 | 1.63 | 1.90 | 1.54 | 1.89 | 1.78 | 1.90 | 1.63 | 1.91 | 1,73 |
| | | SD | 0.24 | 0.31 | 0.18 | 0.22 | 0.31 | 0.32 | 0.27 | 0.35 | 0.30 | 0.21 | 0.38 | 0.23 | 0.30 | 0.35 | 0.29 | 0.25 | 0.33 | 0,26 |
| | p | | 0.07 | 0.19 | 0.83 | 0.52 | 0.10 | 0.83 | 0.66 | 0.34 | 0.55 | 0.55 | 0.99 | 0.78 | 0.13 | 0.13 | 0.10 | 0.34 | 0.18 | 0.28 |
| | t | | 1.92 | 1.34 | 0.21 | 0.66 | 1.74 | -0.22 | -0.45 | 0.97 | -0.62 | 0.61 | 0.01 | -0.28 | -1.59 | -1.59 | -1.73 | -0.98 | -1.40 | 1.11 |
| R-MYO [ms] | S1 | $\bar{x}$ | 20.89 | 21.27 | 20.21 | 18.52 | 23.13 | 18.38 | 18.45 | 20.81 | 19.84 | 20.34 | 21.19 | 19.59 | 18.65 | 22.50 | 18.21 | 17.60 | 20.51 | 18,85 |
| | | SD | 1.84 | 4.19 | 1.98 | 2.36 | 2.82 | 2.51 | 2.60 | 4.37 | 2.22 | 2.86 | 2.96 | 3.11 | 3.24 | 2.93 | 3.04 | 2.93 | 3.29 | 2,79 |
| | S2 | $\bar{x}$ | 20.70 | 19.44 | 19.96 | 18.36 | 22.73 | 18.13 | 18.41 | 19.28 | 19.90 | 19.79 | 19.75 | 19.44 | 18.31 | 21.67 | 18.01 | 17.83 | 19.77 | 19,42 |
| | | SD | 2.59 | 3.67 | 2.81 | 2.94 | 2.75 | 2.96 | 2.81 | 4.11 | 2.52 | 2.74 | 3.81 | 2.86 | 2.60 | 3.51 | 2.41 | 2.99 | 3.50 | 2,95 |
| | p | | 0.50 | < .001 | 0.40 | 0.58 | 0.24 | 0.42 | 0.88 | 0.01 | 0.79 | 0.13 | 0.03 | 0.64 | 0.34 | 0.14 | 0.56 | 0.51 | 0.03 | 0.09 |
| | t | | 0.69 | 3.80 | 0.85 | 0.56 | 1.20 | 0.82 | 0.15 | 2.72 | -0.27 | 1.56 | 2.35 | 0.47 | 0.98 | 1.52 | 0.59 | -0.67 | 2.25 | -1.78 |
| C-MYO [De] | S1 | $\bar{x}$ | 1.23 | 1.28 | 1.21 | 1.11 | 1.37 | 1.11 | 1.11 | 1.26 | 1.19 | 1.20 | 1.25 | 1.16 | 1.11 | 1.32 | 1.09 | 1.04 | 1.23 | 1,12 |
| | | SD | 0.10 | 0.24 | 0.11 | 0.13 | 0.18 | 0.14 | 0.14 | 0.26 | 0.12 | 0.16 | 0.16 | 0.17 | 0.18 | 0.18 | 0.17 | 0.15 | 0.18 | 0,15 |
| | S2 | $\bar{x}$ | 1.22 | 1.17 | 1.19 | 1.11 | 1.35 | 1.10 | 1.11 | 1.17 | 1.19 | 1.17 | 1.18 | 1.15 | 1.10 | 1.30 | 1.08 | 1.07 | 1.19 | 1,15 |
| | | SD | 0.14 | 0.21 | 0.16 | 0.17 | 0.17 | 0.17 | 0.15 | 0.24 | 0.13 | 0.14 | 0.20 | 0.15 | 0.14 | 0.21 | 0.13 | 0.16 | 0.19 | 0,15 |
| | p | | 0.38 | < .001 | 0.44 | 0.80 | 0.32 | 0.58 | 0.77 | 0.01 | 0.89 | 0.11 | 0.08 | 0.81 | 0.46 | 0.50 | 0.70 | 0.27 | 0.11 | 0.13 |
| | t | | 0.90 | 3.76 | 0.78 | 0.25 | 1.02 | 0.56 | 0.30 | 2.75 | -0.14 | 1.68 | 1.86 | 0.24 | 0.75 | 0.68 | 0.40 | -1.14 | 1.66 | -1.56 |

U1-U3, D1-D3, measurement points on the scar; L, R, U, D, direction of measurement, left, right, up, down, respectively; F-MYO, myotonometric frequency, S-MYO, myotonometric stiffness, D-MYO, myotonometric decrement, R-MYO, myotonometric relaxation time, C-MYO, myotonometric creep; S1, measuring session 1; S2, measuring session 2; $\bar{x}$, mean; SD, standard deviation; p, p-value; t, the ratio of the difference.
